# Metal-only Lewis pairs between group 10 metals and Tl(i) or Ag(i): insights into the electronic consequences of Z-type ligand binding†
†Electronic supplementary information (ESI) available: Synthetic and spectroscopic details, crystallographic structure determinations and CSD search results. CCDC 1414413 – 1414422. For ESI and crystallographic data in CIF or other electronic format see DOI: 10.1039/c5sc03104d


**DOI:** 10.1039/c5sc03104d

**Published:** 2015-09-17

**Authors:** Brandon R. Barnett, Curtis E. Moore, Perumalreddy Chandrasekaran, Stephen Sproules, Arnold L. Rheingold, Serena DeBeer, Joshua S. Figueroa

**Affiliations:** a Department of Chemistry and Biochemistry , University of California , San Diego, 9500 Gilman Drive, Mail Code 0358 , La Jolla , CA 92093 , USA . Email: jsfig@ucsd.edu; b Department of Chemistry and Biochemistry , Lamar University , Beaumont , TX 77710 , USA; c School of Chemistry , University of Glasgow , Glasgow G12 8QQ , UK; d Max-Planck-Institute for Chemical Energy Conversion , Stiftstrasse 34-36 , D-45470 , Mülheim an der Ruhr , Germany; e Department of Chemistry and Chemical Biology , Cornell University , Ithaca , New York 14853 , USA

## Abstract

Complexes bearing electron rich transition metal centers, especially those displaying coordinative unsaturation, are well-suited to form reverse-dative σ-interactions with Lewis acids. Herein we demonstrate the generality of zerovalent, group 10 *m*-terphenyl isocyanide complexes to form reverse-dative σ-interactions to Tl(i) and Ag(i) centers. Structural and spectroscopic investigations of these metal-only Lewis pairs (MOLPs) has allowed insight into the electronic consequences of Lewis-acid ligation within the primary coordination sphere of a transition metal center. Treatment of the bis-isocyanide complex, Pt(CNAr^Dipp2^)_2_ (Ar^Dipp2^ = 2,6-(2,6-(i-Pr)_2_C_6_H_3_)_2_C_6_H_3_) with TlOTf (OTf = [O_3_SCF_3_]^–^) yields the Pt/Tl MOLP [TlPt(CNAr^Dipp2^)_2_]OTf (**1**). ^1^H NMR and IR spectroscopic studies on **1**, and its Pd congener [TlPd(CNAr^Dipp2^)_2_]OTf (**2**), demonstrate that the M → Tl interaction is labile in solution. However, treatment of complexes **1** and **2** with Na[BAr^F^_4_] (Ar^F^ = 3,5-(CF_3_)_2_C_6_H_3_) produces [TlPt(CNAr^Dipp2^)_2_]BAr^F^_4_ (**3**) and [TlPd(CNAr^Dipp2^)_2_]BAr^F^_4_ (**4**), in which Tl(i) binding is shown to be static by IR spectroscopy and, in the case of **3**, ^195^Pt NMR spectroscopy as well. This result provides strong evidence that the M → Tl linkages can be attributed primarily to σ-donation from the group 10 metal to Tl, as loss of ionic stabilization of Tl by the triflate anion is compensated for by increasing the degree of M → Tl σ-donation. In addition, X-ray Absorption Near-Edge Spectroscopy (XANES) on the Pd/Tl and Ni/Tl MOLPs, [TlPd(CNAr^Dipp2^)_2_]OTf (**2**) and [TlNi(CNAr^Mes2^)_3_]OTf, respectively, is used to illustrate that the formation of a reverse-dative σ-interaction with Tl(i) does not alter the spectroscopic oxidation state of the group 10 metal. Also reported is the ability of M(CNAr^Dipp2^)_2_ (M = Pt, Pd) to form MOLPs with Ag(i), yielding the complexes [AgM(CNAr^Dipp2^)_2_]OTf (**5**, M = Pt; **6**, M = Pd). As was determined for the Tl-containing MOLPs **1–4**, it is shown that the spectroscopic oxidation states of the group 10 metal in **5** and **6** are essentially unchanged compared to the zerovalent precursors M(CNAr^Dipp2^)_2_. However, in the case of **5** and **6**, the formation of a dative M → Ag σ-bonding interaction facilitates the binding of Lewis bases to the group 10 metal *trans* to Ag, illustrating the potential of acceptor fragments to open up new coordination sites on transition metal complexes without formal, two-electron oxidation.

## Abstract 2

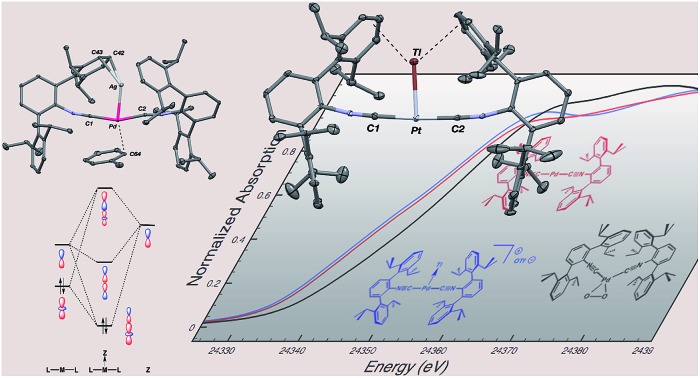
Metal-only Lewis Pairs between Group 10 metals and Tl(i) and Ag(i) have allowed for insight into the electronic consequences of Lewis-acid ligation.

## Introduction

On account of their relatively electropositive nature and ability to act as formal acceptors toward Lewis bases, the transition metals in coordination complexes are traditionally viewed as Lewis acids. Classical “Werner-type” complexes utilize their empty *n*d, as well as (*n* + 1)s and (*n* + 1)p, orbitals to form dative bonds with electron-donor ligands. In the case of highly reduced and electron-rich complexes, the transition metal center may also be capable of exhibiting Lewis basic behavior.^1^ Although this phenomenon was initially invoked for the case of carbonyl metallates acting as Brønsted bases,^2^–^4^ it is now recognized as a central tenet of transition-metal bonding to π-acidic ligands^5^–^7^ as well as an essential component of many oxidative addition mechanisms.^8^–^12^ More recently, the extension of this concept to the binding of various main-group acceptor fragments (Z-type ligands)^13^ in a σ-fashion by electron-rich transition metals has been realized, and the study of such complexes continues to be of intense interest.^14^–^27^

In addition to these examples, a related topic concerning transition metal Lewis basicity is the ability to form dative interactions to another metal center. Judicious ligand design strategies that constrain an electron-rich metal center in close proximity to a coordinatively unsaturated metal fragment has proven to be a reliable approach for engendering metal–metal dative bonding.^28^–^34^ Furthermore, in certain instances, unsupported metal-only Lewis pairs (MOLPs), which do not rely on a ligand buttress, can be generated.^2^,^35^–^39^ The formation of such unsupported metal–metal interactions, while sometimes labile in solution, offers an interesting approach toward tuning the reactivity profiles of low-valent complexes, as the addition of metallic Lewis acids has been shown to enhance the rates of certain catalytic processes.^40^–^43^

While synthetic methods leading to MOLPs and their structural chemistry has advanced, a detailed understanding of how the presence of a metal–metal dative bond affects the electronic properties of the constituent fragments remains of significant interest. It is generally accepted that protonation of a transition metal complex is best viewed as involving a two-electron oxidation of the metal center to give a hydride ligand.^44^ As the electrons involved in the M–H bond have necessarily come from the metal, an increase of its valence by two units is required.^45^ In the case of other main group Lewis acids (*e.g.* boranes), the degree of charge transfer is often not as clear. As such, the adoption and assignment of formalisms to adequately describe the electronic structure of such adducts has been a point of debate in the community.^46^,^47^ Similar ambiguities in the electronic structures of MOLPs exist, although considerably less effort has been put toward uncovering satisfactory electronic descriptors for such compounds.^39^ Despite the fact that X-ray Absorption Near-Edge Spectroscopy (XANES) holds promise in this regard,^30^,^34^ its thus-far limited use in this capacity has not yet led to the development of general principles for properly describing the electronic structures of complexes containing metal–metal dative interactions.

Work from our research group has demonstrated the utility of encumbering *m*-terphenyl isocyanides in stabilizing low-valent and coordinatively unsaturated complexes of late transition metals.^27^,^48^–^55^ Such electron-rich metal centers are prime candidates for acting as Lewis bases toward appropriate Lewis acidic substrates, a concept that has been demonstrated by the heterobimetallics [TlNi(η^4^-COD)(CNAr^Mes2^)_2_]X (X = OTf, BAr^F^_4_),^49^ [TlNi(CNAr^Mes2^)_3_]OTf,^49^ and [TlPd(CNAr^Dipp2^)_2_]OTf (**2**),^50^ as well as by the recently-reported platinum (boryl)iminomethane complex Pt(κ^2^-N,B-^Cy2^BIM)(CNAr^Dipp2^).^27^ In addition, the response of the isocyanide *ν*(C≡N) IR bands to the electron density on the Lewis-basic metal center renders them a convenient spectroscopic reporter on the degree of formal charge transfer upon binding a σ-acceptor fragment.^25^ In this work, we demonstrate the ability of the two-coordinate complexes M(CNAr^Dipp2^)_2_ (M = Pt, Pd)^27^,^50^ to form unsupported metal–metal linkages with Tl(i). Two Tl-containing MOLPs have also been examined by X-ray Absorption Near-Edge Spectroscopy (XANES), illustrating that the spectroscopic oxidation state of the group 10 metal is not affected by its interaction with Tl(i). We also show that the zero-valent platforms M(CNAr^Dipp2^)_2_ (M = Pt, Pd) can form metal-only Lewis pairs with Ag(i), yielding the heterobimetallic salts [AgM(CNAr^Dipp2^)_2_]OTf (**5**, M = Pt; **6**, M = Pd). Spectroscopic and structural investigations provide insight into the nature of the M–Ag interactions in these compounds, and give strong evidence that formation of the M → Ag linkage results in only a marginal degree of metal-to-metal charge transfer. In the case of the Pt variant **5**, further aggregation with additional AgOTf leads to dimeric {[Ag_2_Pt(CNAr^Dipp2^)_2_(η^1^-C_6_H_6_)]_2_(μ-OTf)_2_}(OTf)_2_ (**7**) containing *triangulo*-PtAg_2_ cores. It is shown that binding of one (compounds **5** and **6**) and two (compound **7**) equivalents of Ag(i) results in a sequential increase in the Lewis acidity of the group 10 metal center, thus illustrating how σ-acceptor fragments can be used to rationally tune the properties of electron-rich transition metal complexes.

## Results and discussion

**Scheme 1:**
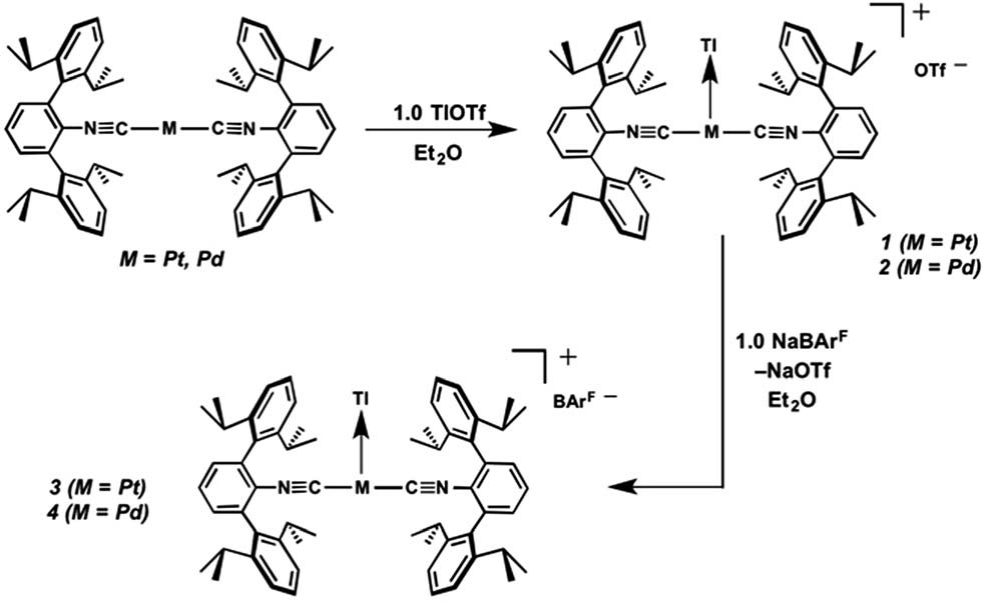


**Fig. 1:**
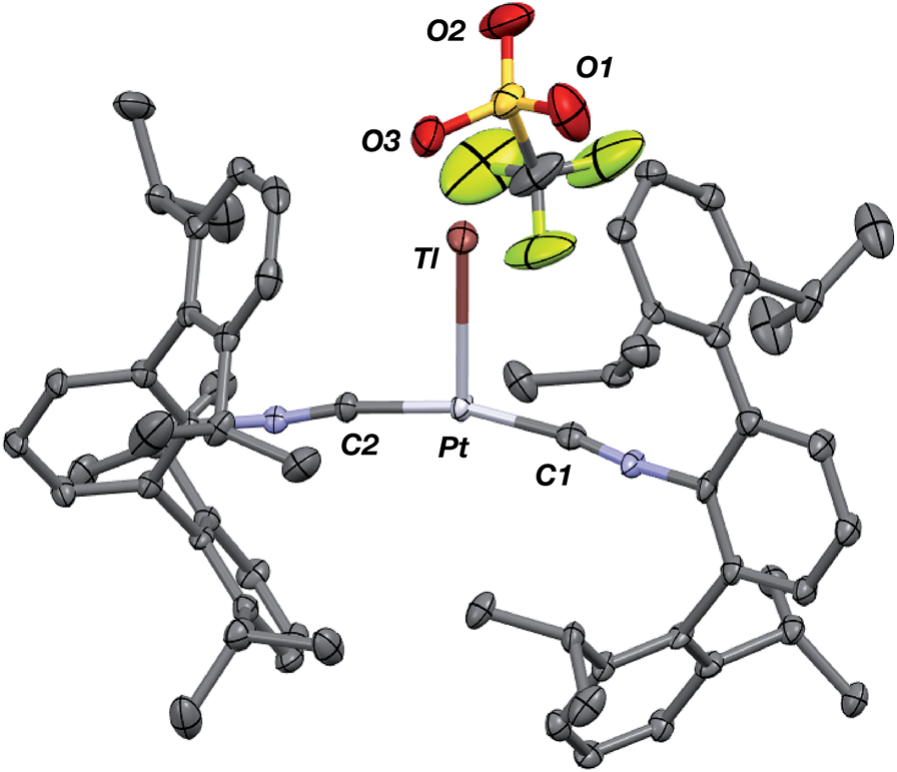
Molecular structure of [TlPt(CNAr^Dipp2^)_2_]OTf (**1**).

Similar to the zero-valent Pd congener, Pd(CNAr^Dipp2^)_2_,^50^ the addition of TlOTf to a solution of Pt(CNAr^Dipp2^)_2_ in Et_2_O yields the unsupported heterobimetallic compound [TlPt(CNAr^Dipp2^)_2_]OTf (**1**) as a yellow microcrystalline solid. Structural characterization of **1** (Scheme 1 and Fig. 1) reveals a T-shaped coordination geometry about Pt, while the Tl center makes long, but non-negligible contacts with the [OTf]^–^ anion (*d*(Tl–O3) = 2.799(5) Å) and the C_aryl_ atoms of the Dipp rings (shortest *d*(Tl–C_aryl_) = 3.355 Å). The presence of a Pt–Tl bonding interaction is apparent given their interatomic separation of 2.8617(3) Å. Importantly, this value is comparable to the most reasonable range for the sum of the covalent radii between Pt and Tl (2.67–2.84 Å),^56^ thereby suggesting that the solid-state structure of **1** does not simply arise from the co-crystallization of Pt(CNAr^Dipp2^)_2_ with TlOTf. While the role of closed-shell metallophilic interactions^57^ cannot be completely discounted, spectroscopic evidence indicates that this interaction is formed by a reverse-dative σ-bond, whereby Pt donates two electrons to an empty 6p orbital on Tl. Analysis of these solutions by FTIR spectroscopy shows a strong *ν*(C≡N) band at 2112 cm^–1^, which is shifted to higher energy relative to those of Pt(CNAr^Dipp2^)_2_ (2065, 2020 cm^–1^),^27^ consistent with a decrease in π-backbonding interactions to the isocyanides as a result of the formation of a Pt → Tl retrodative σ-bonding interaction. A similar blue-shift of this band for the palladium analogue [TlPd(CNAr^Dipp2^)_2_]OTf (**2**) with respect to Pd(CNAr^Dipp2^)_2_ was observed previously.^50^ Surprisingly, bonds between electron-rich, late transition metals (especially third-row metals) and Tl(i) have often been rationalized largely based on metallophilic interactions.^58^–^61^ However, the FTIR spectra of **1** and **2** compared with those of M(CNAr^Dipp2^)_2_ (M = Pt, Pd) provide strong experimental evidence that late-metal-Tl(i) bonds likely contain a substantial dative-bonding component in a manner analogous to that seen for complexes bearing main-group Z-type ligands.^23^–^25^

**Fig. 2:**
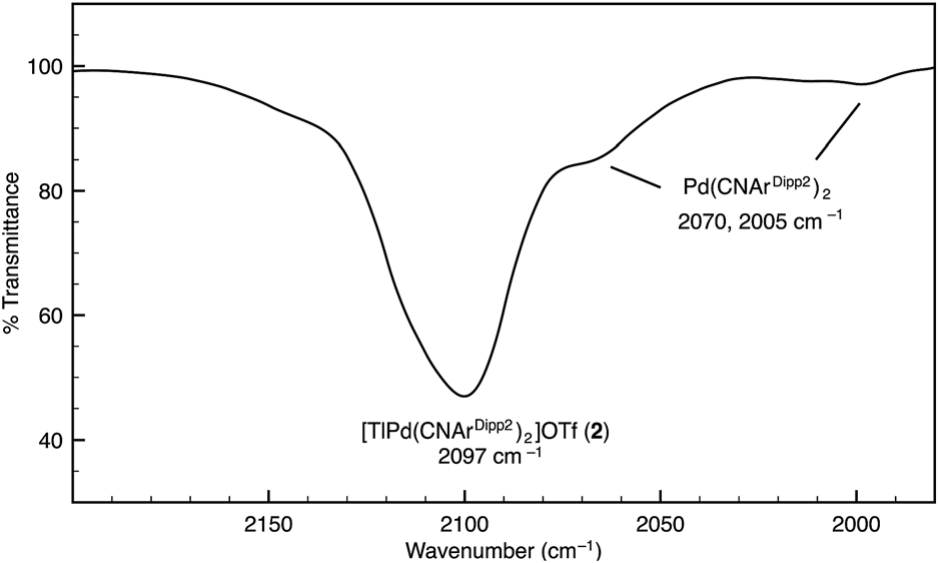
FTIR spectrum (*ν*(C≡N) region) of [TlPd(CNAr^Dipp2^)_2_]OTf (**2**) showing the presence of Pd(CNAr^Dipp2^)_2_. Conditions: C_6_D_6_, 20 °C, KBr windows.

Although [TlPt(CNAr^Dipp2^)_2_]OTf (**1**) gives rise to a sharp set of ^1^H and ^13^C{^1^H} NMR resonances in benzene-*d*_6_, other spectroscopic data suggest that the metal–metal interaction is labile in solution. While the IR absorption bands of Pt(CNAr^Dipp2^)_2_ are not apparent in the IR spectrum of **1**, it is important to note that *ν*(C≡N) bands corresponding to Pd(CNAr^Dipp2^)_2_ are readily observable as a minor component in the IR spectrum of [TlPd(CNAr^Dipp2^)_2_]OTf (**2**) in C_6_D_6_ solution, thereby suggesting the presence of an equilibrium between bound and unbound Tl(i) (Fig. 2). In addition, extended scanning failed to locate the ^195^Pt NMR^62^ resonance for the platinum analogue [TlPt(CNAr^Dipp2^)_2_]OTf (**1**). We suggest that this observation is indicative of lability in the Pt–Tl interaction on the NMR timescale, resulting in a broadening of this resonance that obviates its detection at room temperature.

**Fig. 3:**
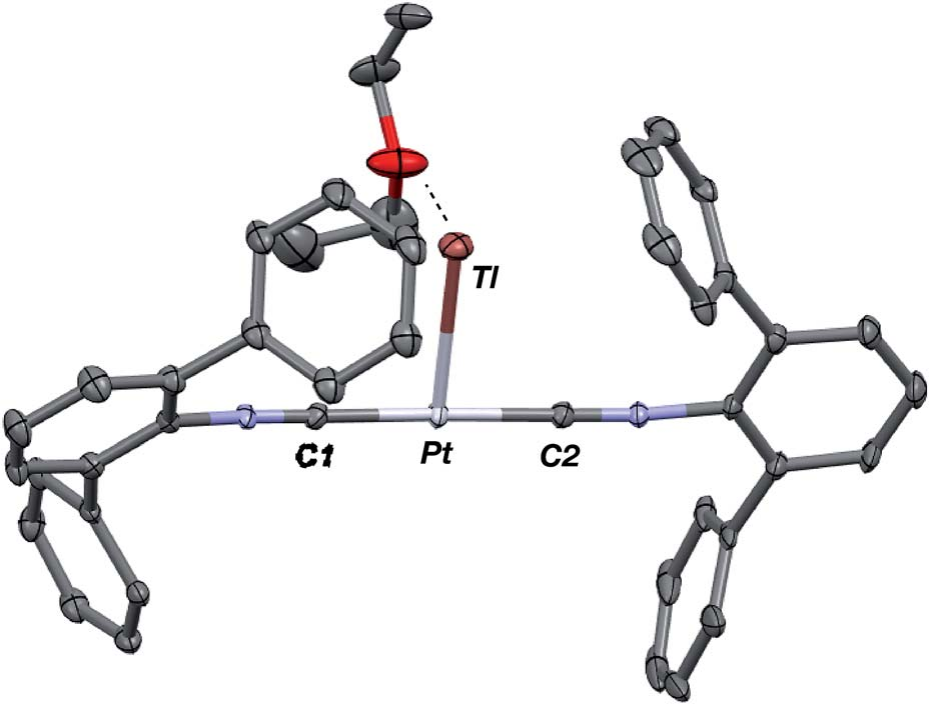
Molecular structure of [(Et_2_O)TlPt(CNAr^Dipp2^)_2_]BAr^F^_4_ (**3**(Et_2_O)). Isopropyl groups and BAr^F^_4_ counterion have been omitted for clarity. The Pd congener **4**(Et_2_O) is isostructural.

**Fig. 4:**
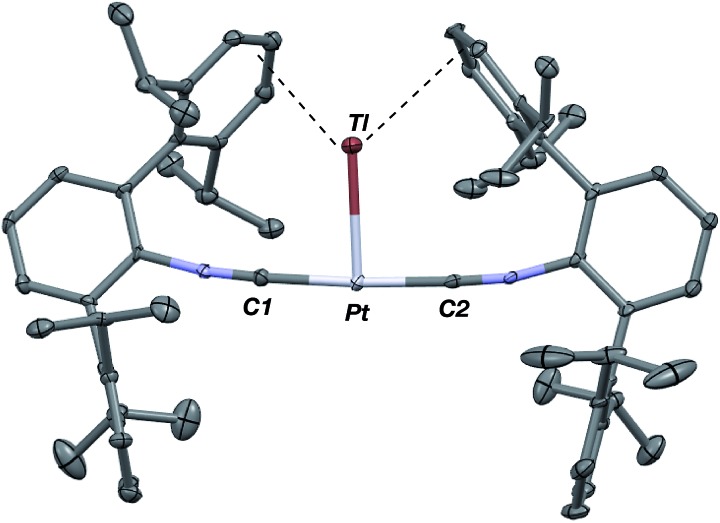
Molecular structure of [TlPt(CNAr^Dipp2^)_2_]BAr^F^_4_ (**3**). BAr^F^_4_ counterion has been omitted for clarity.

**Fig. 5:**
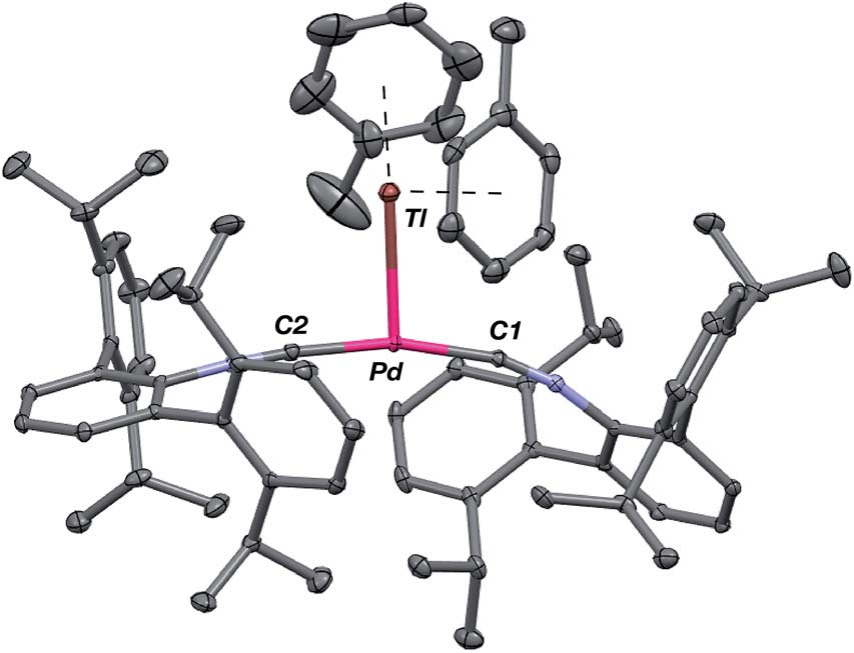
Molecular structure of [(η^6^-Tol)_2_TlPd(CNAr^Dipp2^)_2_]BAr^F^_4_ (**4**(Tol)_2_). BAr^F^_4_ counterion has been omitted for clarity.

As the lability of unsupported M–Tl linkages has been observed to display a dependence on counteranion identity,^58^ we sought to explore the behavior of [TlM(CNAr^Dipp2^)_2_]^+^ (M = Pt, Pd) when accompanied by a traditionally non-coordinating anion. Addition of an Et_2_O solution of NaBAr^F^_4_ (BAr^F^_4_ = [B(3,5-(CF_3_)_2_C_6_H_3_)_4_]^–^) to **1** or **2** results in precipitation of NaOTf and smooth formation of [(Et_2_O)TlM(CNAr^Dipp2^)_2_]BAr^F^_4_ (M = Pt (**3**(Et_2_O)), Pd (**4**(Et_2_O))) following crystallization from Et_2_O (Fig. 3). Structural determinations of **3**(Et_2_O) and **4**(Et_2_O) reveal discreet cation–anion pairs (two independent pairs per asymmetric unit). While no contact between the Tl center and the [BAr^F^_4_]^–^ anion is evident in the solid state, the Tl center is bound to a molecule of Et_2_O in both complexes (average *d*(Tl–O) = 2.760(3) Å (**3**) and 2.729(3) Å (**4**)). Furthermore, as noted for **1**, long-range contacts (*ca.* 3.4 Å) between Tl and several C_aryl_ atoms of the flanking Dipp rings are apparent. The Tl-bound ether molecules are easily liberated from crystalline samples upon prolonged exposure to vacuum (∼100 mTorr) as assayed by ^1^H NMR spectroscopy. Subsequent crystallization of **3** from toluene produces solvent-free [TlPt(CNAr^Dipp2^)_2_]BAr^F^_4_ (**3**, Fig. 4), which contains Tl-(η^2^-arene) interactions with the flanking Dipp rings (*d*(Tl–C) = 3.188–3.616 Å).^63^ In the case of **4**, cooling a saturated toluene solution to –35 °C yields [(η^6^-Tol)_2_TlPd(CNAr^Dipp2^)_2_]BAr^F^_4_ (**4**(Tol)_2_, Fig. 5), which displays two toluene molecules bound in an η^6^-fashion to Tl (*d*(Tl–C) = 3.192–3.589 Å). Although the Tl–C_arene_ distances in **3** and **4**(Tol)_2_ are seemingly long, they are well in line with other structurally characterized examples of Tl-arene π-complexes.^60^,^64^–^67^

Examination of the solid-state and solution-phase behaviour of **1–4** reveals that replacement of the triflate anion with [BAr^F^_4_]^–^ has important ramifications for the lability of the M → Tl linkage. In both solvates of **3** and **4**, the M–Tl distance is significantly contracted relative to **1** and **2** (Table 1), consistent with an increase in the degree of M → Tl donation. As the triflate anion likely stabilizes the Tl center through contact ion pairing, replacement of [OTf]^–^ with neutral Et_2_O or arene donors appears to only partially compensate for the loss of this ionic association. Accordingly, in an attempt to recoup some of this stabilization, we contend that the degree of M → Tl σ-donation is increased. This notion is supported by the progression of the *ν*(C≡N) bands in **3** (2121 cm^–1^) and **4** (2116 cm^–1^) to higher energies relative to **1** and **2**, as the increased withdrawal of electron density from the group 10 metal by Tl serves to attenuate backbonding interactions with the isocyanide ligands. Importantly, and in contrast to the triflate salt [TlPd(CNAr^Dipp2^)_2_]OTf (**2**), the solution FTIR spectra of **3** and **4** in benzene-*d*_6_ are devoid of *ν*(C≡N) features corresponding to M(CNAr^Dipp2^)_2_, signalling that Tl(i) dissociation in benzene can be significantly inhibited by the use of the weakly coordinating [BAr^F^_4_]^–^ anion. Interestingly, this replacement also allows for detection of the ^195^Pt NMR resonance of [TlPt(CNAr^Dipp2^)_2_]BAr^F^_4_ (**3**), which appears as a doublet with well-resolved coupling to ^205^Tl (*δ* = –3802 ppm, ^1^*J*_Pt,Tl_ = 11.2 kHz).^68^ This resonance is shifted significantly downfield respective to that of Pt(CNAr^Dipp2^)_2_ (*δ* = –5993 ppm (s), C_6_D_6_), further suggestive of decreased electron density at the Pt center upon coordination of Tl(i).^69^,^70^ However, it is also important to note that dissolution of **1–4** in THF results in complete dissociation of the Tl(i) center and formation of M(CNAr^Dipp2^)_2_, according to FTIR spectroscopy. This result, which was similarly observed in the case of [TlNi(CNAr^Dipp2^)_3_]OTf,^54^ serves as a reminder of the weak dissociation energies inherent in most unsupported metal–metal dative bonds, as dissolution in solvents of moderate coordinating strength is sufficient to completely disrupt this interaction.

**Table 1:** Selected bond distances (X-ray structure) of Pt(CNAr^Dipp2^)_2_, Pd(CNAr^Dipp2^)_2_, and their Tl-containing MOLPs

Complex	*d*(M–C_iso_)	*d*(M–Tl)
Pt(CNAr^Dipp2^)_2_^*a*^	1.906(3) Å	—
Pd(CNAr^Dipp2^)_2_^*b*^	1.930(4) Å	—
**1**	1.918(5) Å	2.8617(3) Å
**2** ^*b*^	1.951(7) Å	2.855(1) Å
**3**(Et_2_O)^*c*^	1.922(4) Å	2.7710(4) Å
**4**(Et_2_O)^*c*^	1.958(4) Å	2.7481(5) Å
**3**	1.933(8) Å	2.7659(5) Å
**4**(Tol)_2_	1.965(3) Å	2.7770(2) Å

^*a*^Data from ref. 27.

^*b*^Data from ref. 50.

^*c*^One of the two crystallographically independent molecules in the X-ray structures of **3**(Et_2_O) and **4**(Et_2_O) contain two-site disorder of the Tl atom. We have used the major component of this disorder to calculate the average *d*(M–Tl).

**Fig. 6:**
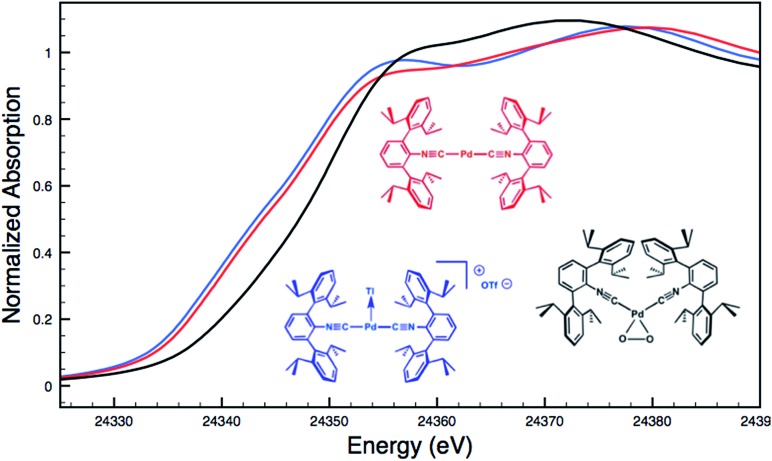
Comparative Pd *K*-edge XANES spectra of Pd(CNAr^Dipp2^)_2_ (red), [TlPd(CNAr^Dipp2^)_2_]OTf (**2**, blue), and Pd(η^2^-O_2_)(CNAr^Dipp2^)_2_ (black).

Although limited experimental techniques are capable of probing metal–metal dative interactions, X-ray Absorption Near-Edge Spectroscopy (XANES)^71^ has begun to find an important use in this regard.^30^,^34^ Importantly, its utility lies in its ability to decipher the spectroscopic oxidation states of the metals involved in a given bonding interaction. In order to assess the degree of charge transfer inherent in the formation of a reverse-dative σ-interaction to Tl(i), Pd *K*-edge XANES was carried out on the palladium–thallium adduct [TlPd(CNAr^Dipp2^)_2_]OTf (**2**, Fig. 6). While neither the Pd *K*-edge spectra of **2** nor that of Pd(CNAr^Dipp2^)_2_ display a discernable pre-edge feature, both exhibit nearly identical energies for the rising edge of the XANES region. In comparison, the rising edge energy of the Pd(ii) peroxo complex^50^,^72^ Pd(η^2^-O_2_)(CNAr^Dipp2^)_2_ is shifted to higher energy by *ca.* 4.0 eV relative to that of Pd(CNAr^Dipp2^)_2_ and **2**. Despite their differing geometries, the rising edge transition for each of these three Pd complexes should involve the promotion of a core 1s electron to a 5p orbital that is relatively unperturbed by ligand field effects. Accordingly, the marked shift of the rising edge to higher energy for Pd(η^2^-O_2_)(CNAr^Dipp2^)_2_ can be reasonably attributed to the presence of an oxidized Pd center relative to that found in Pd(CNAr^Dipp2^)_2_ or **2**. However, the near-identical rising edge energies observed for Pd(CNAr^Dipp2^)_2_ and **2** strongly reflect that Tl(i) binding to an electron rich Pd center does not result in a formal oxidative event.

**Fig. 7:**
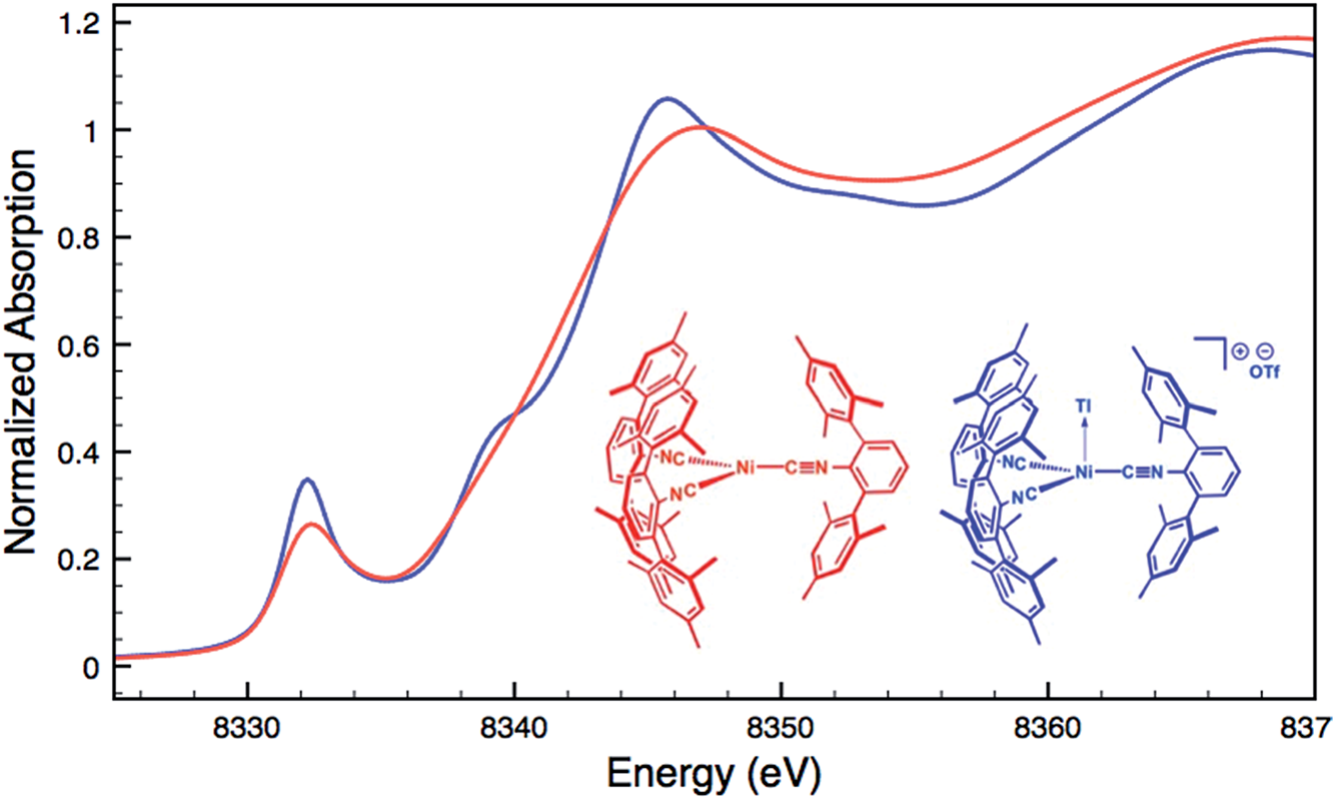
Comparative Ni *K*-edge XANES spectra of Ni(CNAr^Mes2^)_3_ (red) and [TlNi(CNAr^Mes2^)_3_]OTf (blue).

For an additional comparison, XANES measurements were carried out on the binary nickel tris-isocyanide complex Ni(CNAr^Mes2^)_3_ and its adduct with Tl(i), [TlNi(CNAr^Mes2^)_3_]OTf.^49^ Despite the unambiguous d^10^ configuration of Ni(CNAr^Mes2^)_3_, its Ni *K*-edge absorption spectrum (Fig. 7) displays a prominent pre-edge feature, which is likely the result of a 1s to isocyanide π* transition. The analogous absorption band for [TlNi(CNAr^Mes2^)_3_]OTf occurs at an identical energy, again signalling that the formation of a reverse-dative M → Tl σ-interaction does not result in significant formal charge transfer from the group 10 metal.

The fact that neither Pd(CNAr^Dipp2^)_2_ nor Ni(CNAr^Mes2^)_3_ undergo significant charge transfer *via* the formation of a reverse-dative σ-interaction to Tl(i) suggests some important guidelines regarding the proper formalisms that should be used to describe such MOLPs. Although Tl(i) can exhibit Lewis basic properties under extraordinary conditions,^73^ the stabilization of its 6s^2^ “inert pair” due to relativistic effects^74^ should render it a very weak 2e^–^ donor. As such, the electrons involved in a covalent interaction between an electron-rich transition metal and Tl(i) center will most plausibly be supplied by the former, meaning that the valence count of the transition metal must necessarily increase by two units.^45^ However, this interaction should *not* be described as effecting a two-unit increase in the formal oxidation state of the transition metal, as such an event would be readily apparent in the comparative XANES spectra of M(CNR)_*n*_ and [TlM(CNR)_*n*_]^+^ complexes. This conclusion is further supported by the modest changes in the FTIR *ν*(C≡N) energies between the neutral parent compounds and their Tl(i) adducts (*ca.* 50 cm^–1^). For comparison, the Pd(ii) and Ni(ii) complexes *trans*-PdCl_2_(CNAr^Dipp2^)_2_ and *trans*-NiCl_2_(CNAr^Mes2^)_2_ display IR *ν*(C≡N) bands that are blue-shifted by *ca.* 200 cm^–1^ relative to Pd(CNAr^Dipp2^)_2_ and Ni(CNAr^Mes2^)_3_.^49^,^75^

**Fig. 8:**
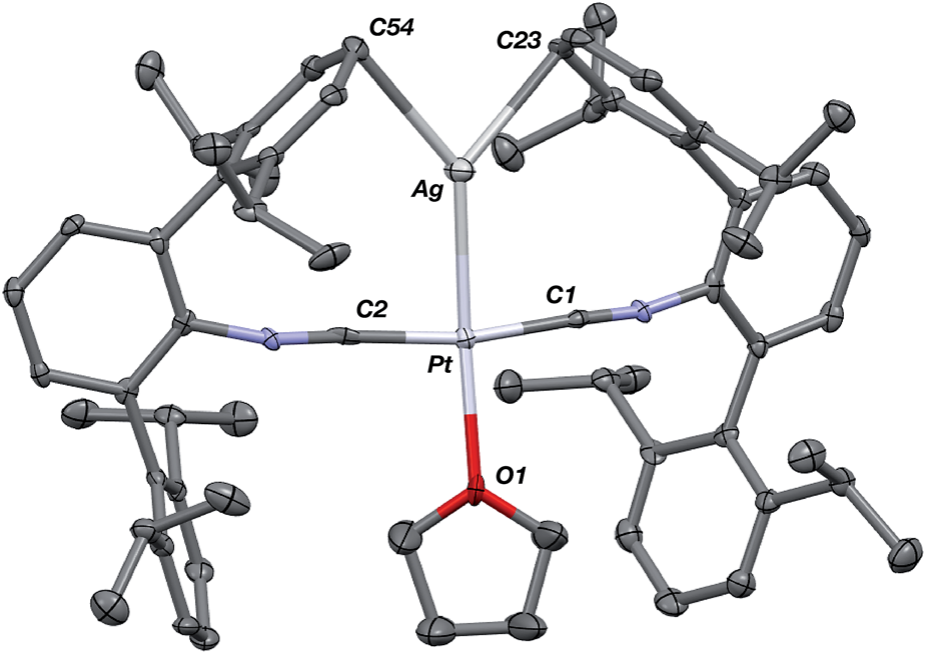
Molecular structure of [AgPt(CNAr^Dipp2^)_2_(THF)]OTf (**5**(THF)). The Pd congener **6**(THF) is isostructural. Triflate counterion has been omitted for clarity.

**Fig. 9:**
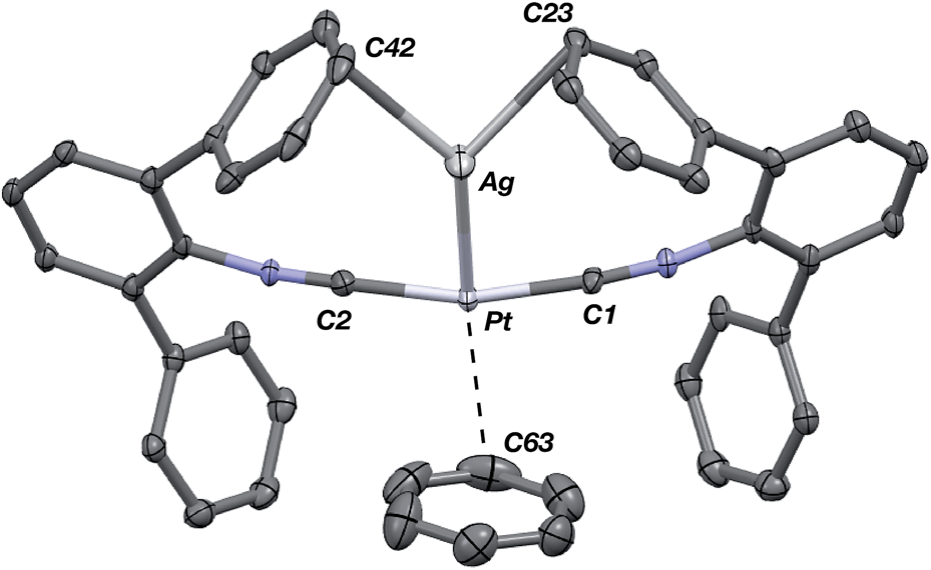
Molecular structure of [AgPt(CNAr^Dipp2^)_2_(η^1^-C_6_H_6_)]OTf (**5**(C_6_H_6_)). Flanking isopropyl groups and the triflate counter ion have been omitted for clarity.

**Fig. 10:**
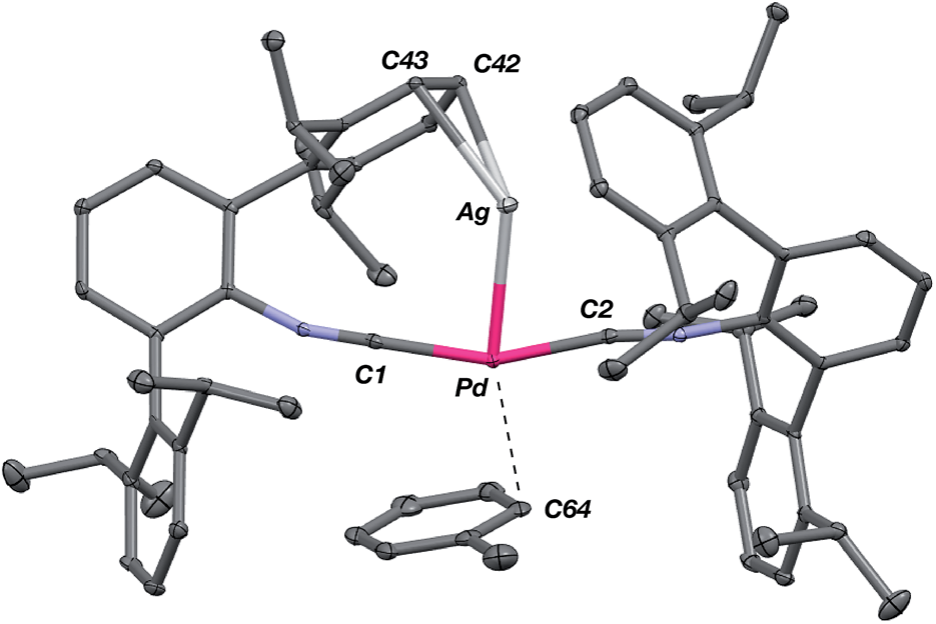
Molecular structure of [AgPd(CNAr^Dipp2^)_2_(η^1^-C_7_H_8_)]OTf (**6**(C_7_H_8_)). Triflate counterion has been omitted for clarity.

**Scheme 2:**
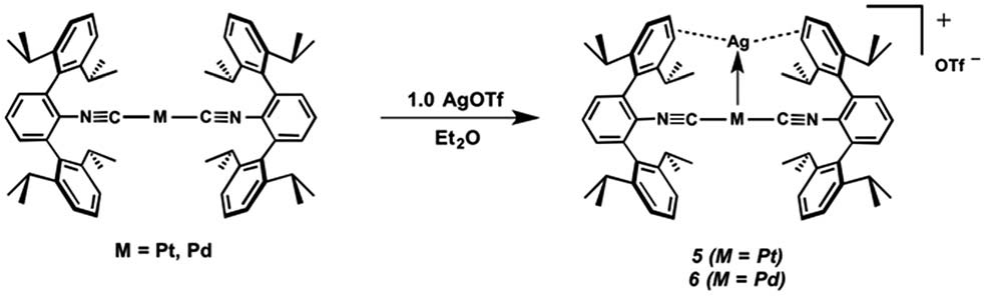


The abilities of M(CNAr^Dipp2^)_2_ (M = Pt, Pd) to act as the basic components of metal-only Lewis pairs can also be extended to Lewis acidic Ag(i) centers.^76^ Treatment of Pt(CNAr^Dipp2^)_2_ with AgOTf in Et_2_O results in precipitation of the heterobimetallic salt [AgPt(CNAr^Dipp2^)_2_]OTf (**5**) as a yellow powder. Attempts to synthesize the palladium analogue [AgPd(CNAr^Dipp2^)_2_]OTf (**6**) in the same fashion results in the formation of metallic mirrors and free CNAr^Dipp2^. However, performing the synthesis at reduced temperatures (*ca.* –100 °C) allows for **6** to be precipitated from solution as a pale yellow powder in modest yields (Scheme 2). Crystallization of **5** or **6** from THF/(TMS)_2_O at –35 °C yields *trans*-[AgM(CNAr^Dipp2^)_2_(THF)]OTf (**5**(THF), M = Pt; **6**(THF), M = Pd), where a molecule of THF is bound to the group 10 metal *trans* to the coordinated Ag center (Fig. 8). The M–O_THF_ distances in **5**(THF) (2.366(5) Å) and **6**(THF) (2.326(7) Å) are long relative to Pd and Pt etherate complexes reported in the Cambridge Structural Database,^77^ thereby suggesting an attenuated interaction of THF with the group 10 metal center. Indeed, the ^1^H NMR spectra obtained from crystalline **5**(THF) and **6**(THF) in C_6_D_6_ show sharp peaks occurring at the expected chemical shift values for free THF.^78^ Further, prolonged exposure of crystalline samples to vacuum (∼100 mTorr) successfully liberates all THF as analyzed by ^1^H NMR spectroscopy. Subsequent recrystallization of these samples from Et_2_O/C_6_H_6_ (**5**) or *n*-hexane/toluene (**6**) yields **5**(C_6_H_6_) and **6**(C_7_H_8_) (Fig. 9 and 10), which display η^1^-C_arene_ interactions between the group 10 metal and arene solvent in the position *trans* to Ag (for **5**, *d*(Pt–C63 = 2.885(7) Å); for **6**, *d*(Pd–C64) = 2.496(3)). These interactions are notable given the likely intermediacy of transient π-complexes^79^ in the C–H activation of arenes by electrophilic group 10 complexes.^80^,^81^

The M–Ag bond lengths in both solvates of **5** and **6** are among the shortest reported in the Cambridge Structural Database (see Table 2 and ESI S1†). As was seen for the M–Tl adducts above, the metal–metal interactions in **5** and **6** can be rationalized *via* M → Ag σ-donation, a notion that is supported by the increase in isocyanide *ν*(C≡N) stretching frequencies relative to M(CNAr^Dipp2^)_2_ (M = Pt, Pd) upon coordination of Ag(i) (*ν*(C≡N) = 2094 cm^–1^ (**5**); 2082 cm^–1^ (**6**)). An examination of the solid state structures of **5** and **6** also implicates a role of the flanking Dipp aryl rings in buttressing the M–Ag linkage through π-type interactions. Interestingly, these contacts are reflected in the room temperature ^1^H NMR spectra of **5** and **6** (measured in C_6_D_6_), for which the resonances corresponding to the Dipp aryl protons are broadened and shifted downfield by *ca.* 0.2 ppm relative to those typically observed for diamagnetic mononuclear complexes containing the CNAr^Dipp2^ ligand. It is also notable that the different solvates of both **5** and **6** display a square-planar coordination environment around the group 10 metal. While these geometries are certainly reminiscent of Pt(ii) and Pd(ii), it is critical to note that the progression of the IR *ν*(C≡N) stretching frequencies to higher energies upon binding of Ag(i) is quite modest and actually less than that seen for Tl(i). This observation serves to suggest that similar bonding descriptions laid out above for Tl-containing **1–4** can be extended to **5** and **6**. While the use of electrons from the group 10 metal to form a covalent interaction with Ag requires an increase of two valence units,^45^ minimal charge transfer to Ag occurs. As such, these M/Ag MOLPs should not be described as containing formal M(ii) centers (M = Pt, Pd).

**Table 2:** Selected bond distances (X-ray structure) of Ag-containing heterobimetallic complexes

Complex	*d*(M–C_iso_)	*d*(M–Ag)
**5**(THF)	1.926(8) Å	2.6299(6) Å
**6**(THF)	1.941(9) Å	2.6303(9) Å
**5**(C_6_H_6_)	1.916(4) Å	2.6463(5) Å
**6**(C_7_H_8_)	1.950(3) Å	2.6112(4) Å

**Fig. 11:**
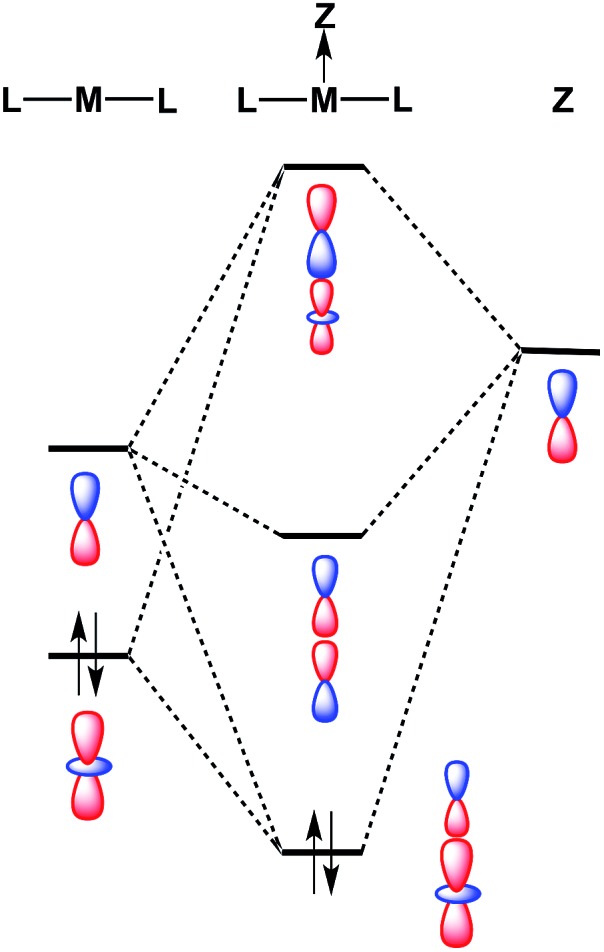
Molecular orbital diagram for a transition metal (**M**) bound to a σ-acceptor fragment (**Z**), showing how the LUMO of the resulting adduct can be stabilized with respect to the acceptor-free complex.

Despite the fact that formation of a M–Ag bonding interaction does not result in a formal oxidative event at Pt/Pd, it is remarkable that the Ag-containing heterobimetallics **5** and **6** will bind THF and arene molecules at the group 10 metal center in the solid state, whereas the zero-valent precursors M(CNAr^Dipp2^)_2_ (M = Pt, Pd) do not. Furthermore, Pt(CNAr^Dipp2^)_2_ and Pd(CNAr^Dipp2^)_2_ do not participate in addition reactions with stronger σ-donors (*e.g.* phosphines) to form species of the type ML(CNAr^Dipp2^)_2_, as the attempted syntheses of such compounds has led invariably to isocyanide dissociation and/or decomposition. While the ability of **5** and **6** to bind an additional Lewis base may be partly attributable to increased positive charge on the complexes, molecular orbital considerations provide a basis for enhanced Lewis acidity at the group 10 metal center of these MOLPs specifically. It has been suggested previously that coordination of a Z-type acceptor ligand to a square-planar d^8^ complex should result in enhanced affinity for Lewis bases at the open coordination site *trans* to the acceptor.^82^ Similarly, formation of a reverse-dative σ-interaction by M(CNAr^Dipp2^)_2_ (nominally from the *n*d_*z*_^2^ orbital) to an acceptor may have a stabilizing effect on the coaxial empty (*n* + 1)p_*z*_ orbital of the group 10 metal (Fig. 11). While such stabilization may not be drastic, it is plausible that such effects could promote the binding of Lewis bases at a coordination site *trans* to the acceptor, resulting in square-planar [AgML_2_L′]^+^-type species. Similar behavior was observed by Peters in the trigonal-pyramidal Pt salt [(SiP^Ph^_3_)Pt]BAr^F^_4_,^83^ (SiP^Ph^_3_ = (2-Ph_2_PC_6_H_4_)_3_Si) for which the crystal structure shows a molecule of toluene bound in the apical position *trans* to the silyl group. As silyl ligands can be viewed in certain systems as silylium Lewis acids,^84^ the binding of an arene molecule may be a result of Pt-to-Si σ-donation, thereby in effect enhancing the Lewis acidic nature of the Pt complex. In addition, similar phenomena have been observed by Gabbaï for a Hg(ii) complex^85^ and by Berry for a bimetallic Mo_2_ system.^86^ In these examples, association of a Z-type fragment was shown to increase Lewis acidity at the coordination site *trans* to the acceptor ligand. However, to our knowledge, the MOLPs derived from M(CNAr^Dipp2^)_2_ (M = Pt, Pd) represent unique cases where Z-ligand-promoted Lewis acidity has been unambiguously observed for mononuclear transition metal complexes. Importantly, these observations highlight the ability of σ-acceptor ligands to open up a previously unavailable coordination site on a transition metal center without effecting a formal oxidative event. Furthermore, the observation that the Ag-containing complexes **5** and **6** bind solvent molecules at the group 10 metal center, while the Tl-containing complexes **1** and **2** exhibit binding at the Tl center, is likely attributable to the greater electronegativity of Ag relative to Tl.^87^ As stabilization of the empty p_*z*_ orbital on the group 10 metal by a bound Lewis acid is expected to be marginal at best, Lewis acids possessing greater group electronegativity may be expected to more effectively stabilize this orbital and render it accessible to an exogenous Lewis base.

**Fig. 12:**
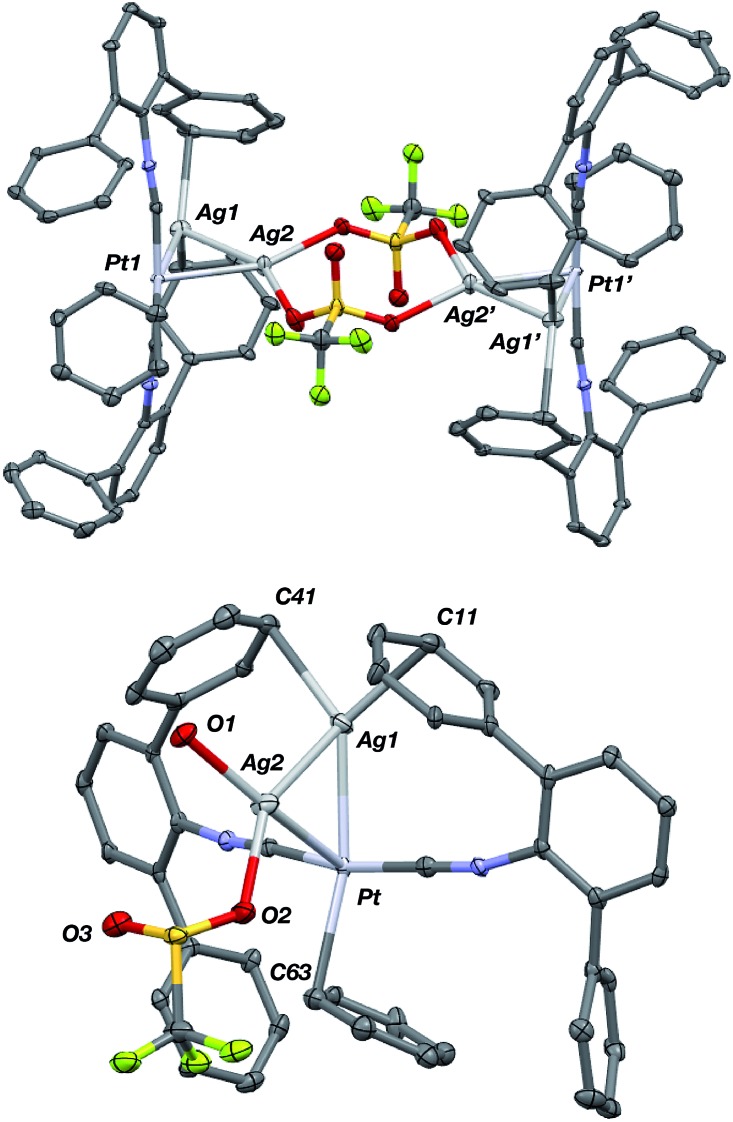
Molecular structure of the cationic portion of {[Ag_2_Pt(CNAr^Dipp2^)_2_(η^1^-C_6_H_6_)]_2_(μ-OTf)_2_}(OTf)_2_ (**7**). Top: Full view of the centro-symmetric dication. Bottom: Truncated view of one *triangulo*-Ag_2_Pt core. In both views, isopropyl groups and non-coordinating triflate anions have been removed for clarity.

Although [AgPt(CNAr^Dipp2^)_2_]OTf (**5**) contains one acceptor fragment bound to platinum, its Pt–Ag unit can accommodate another equivalent of Ag(i). Stirring [AgPt(CNAr^Dipp2^)_2_]OTf (**5**) and equimolar AgOTf in THF followed by crystallization from benzene/THF (20 : 1) yields {[Ag_2_Pt(CNAr^Dipp2^)_2_(η^1^-C_6_H_6_)]_2_(μ-OTf)_2_}(OTf)_2_ (**7**) as determined by X-ray diffraction. Attempts to synthesize a palladium analogue from [AgPd(CNAr^Dipp2^)_2_]OTf (**6**) resulted only in decomposition. The solid-state structure of **7** (Fig. 12) revealed a centro-symmetric dimer composed of *triangulo*-PtAg_2_ cores (average *d*(Pt–Ag) = 2.6843(6) Å; *d*(Ag–Ag) = 2.7684(8) Å) bridged by two triflate groups. Consistent with the coordination of an additional Lewis acid to the Pt–Ag unit in **5**, the isocyanide stretching frequencies of **7** are shifted to higher energies (2132, 2169 cm^–1^, KBr pellet) compared to **5**. In the solid state, the platinum centers in **7** also feature η^1^-C-bound benzene molecules *trans* to one of the silver atoms as seen in **5**(C_6_H_6_). Interestingly however, the Pt–C_benzene_ distance in **7** (*d*(Pt–C1) = 2.529(7) Å) is significantly contracted relative to that in **5**(C_6_H_6_), a further indication of an increase in the Lewis acidity in the Pt center in **7** promoted by the presence of a second Ag center. It is also important to note that relative to complex **5**, the second Ag atom in **7** (*i.e.* Ag(2), Fig. 12) can best be described as occupying the axial position of a nominally square-planar Pt center. As the binding of Lewis acids to the axial position square planar Pt(ii) centers is known,^88^–^92^ complex **7** provides additional evidence that the presence of one Z-type ligand effectively results in the formation of a divalent Pt center capable of binding a second Z-type ligand. However, this electronic modulation occurs without formal oxidation of the Pt center.

## Conclusions

Zero-valent binary *m*-terphenyl isocyanide complexes of Pt and Pd are excellent candidates for acting as the Lewis basic component of metal-only Lewis pairs (MOLPs). In addition to stabilizing low oxidation states, the steric encumbrance of the *m*-terphenyl isocyanide ligand promotes coordinative unsaturation, yielding electron-rich metal centers that can accommodate an exogenous Lewis acid in the primary coordination sphere. Furthermore, the IR *ν*(C≡N) resonances provide a convenient handle to assess the degree of group 10 metal σ-donation in these complexes. In this work, it has been demonstrated that Pt(CNAr^Dipp2^)_2_ and Pd(CNAr^Dipp2^)_2_ can form discreet and unsupported adducts with Tl(i). Although these bonding interactions are not particularly robust, use of the weakly coordinating anion BAr^F^_4_^–^ diminishes the lability of the M–Tl bond in non-coordinating solvents. Analysis of two M → Tl adducts by XANES spectroscopy provides compelling evidence that any degree of formal charge transfer inherent in these metal–metal interactions is minimal, and that therefore no formal oxidative event takes place upon binding of Tl(i). The ability of Pt(CNAr^Dipp2^)_2_ and Pd(CNAr^Dipp2^)_2_ to form Lewis pairs with Ag(i) has also been demonstrated, with FTIR spectroscopy and X-ray crystallography suggesting, again, that no significant charge transfer to Ag occurs in the adducts. Despite this fact, the binding of Ag(i) activates the group 10 metal toward the ligation of Lewis bases *trans* to the Ag acceptor, thus highlighting how σ-acceptor ligands can be utilized to tune the reactivity profiles of electron-rich transition metal complexes. The Pt/Ag MOLP [AgPt(CNAr^Dipp2^)_2_]OTf (**5**) can also accommodate an additional equivalent of AgOTf to form dimeric **7**, which further increases the Lewis acidity of the Pt center. These results indicate that the presence of a reverse-dative σ-interaction can activate the coordination site *trans* to it for binding of Lewis bases despite the high *trans* influence exhibited by Z-type ligands.^24^,^25^ Such modulation can be thought of as a novel type of cooperative effect between a Lewis acid and Lewis base, whereby the former alters the reactivity profile of the electron rich metal without directly participating in the reaction with an incoming substrate. A more detailed understanding of the possibilities afforded by such cooperative effects is currently being pursued in our laboratory.

## Supplementary Material

Supplementary information

Crystal structure data
