# Supplementary material for: Metal-only Lewis pairs between group 10 metals and Tl(i) or Ag(i): insights into the electronic consequences of Z-type ligand binding
Source: Chem Sci. 2015 Sep 17;6(12):7169–78. doi: 10.1039/c5sc03104d (PMC5951196; doi:10.1039/c5sc03104d)
Supplement: Supplementary file 1 [file SC-006-C5SC03104D-s001.pdf]

Electronic Supplementary Material (ESI) for Chemical Science.
This journal is © The Royal Society of Chemistry 2015

Supporting Information for the Paper Entitled:

Metal-Only Lewis Pairs Between Group 10 Metals and Tl(I) or Ag(I): Insights in the Electronic Consequences of Z-Type Ligand Binding

Brandon R. Barnett[a], Curtis E. Moore[a], Perumalreddy Chandrasekaran[b], Stephen Sproules[c], Arnold L. Rheingold[a], Serena DeBeer[d,e], and Joshua S. Figueroa*[a]

[a]*Department of Chemistry and Biochemistry, University of California, San Diego, 9500 Gilman Drive Mail Code 0358, La Jolla CA, 92093-0358.*

[b]*Department of Chemistry and Biochemistry, Lamar University, Beaumont, TX 77710.*

[c]*School of Chemistry, University of Glasgow, Glasgow G12 8QQ, UK.*

[d]*Max-Planck-Institute for Chemical Energy Conversion, Stiftstrasse 34-36, D-45470 Mülheim an der Ruhr, Germany*

[e]*Department of Chemistry and Chemical Biology, Cornell University, Ithaca, NY 14853, United States*

Email: jsfig@ucsd.edu

Contents

S1. Synthetic Procedures and Characterization Data……………….……….S-2

S2. CSD Search Results……………...…………………..………………...…S-5

S3. Crystallographic Structure Determinations………………………..…..S-8

S4. References…………………………………………………………….…....S-19

S1. Synthetic Procedures and Characterization Data

S1.1. General considerations. All manipulations were carried out under an atmosphere of purified dinitrogen using standard Schlenk and glovebox techniques. Unless otherwise stated, reagent-grade starting materials were purchased from commercial sources and either used as received or purified by standard procedures.[1] Solvents were dried and deoxygenated according to standard procedures.[2] Benzene-d_6 (Cambridge Isotope Laboratories) was distilled from NaK alloy/benzophenone and stored over activated 4 Å molecular sieves for 2 d prior to use. Celite 405 (Fisher Scientific) was dried under vacuum (24 h) at a temperature above 250 ˚C and stored in the glovebox prior to use. KBr (FTIR grade from Aldrich) was stirred overnight in anhydrous THF, filtered and dried under vacuum (24 h) at a temperature above 250 ˚C and stored in the glovebox prior to use. $Pt(CNAr^{Dipp2})_2$,[3] $[TlPd(CNAr^{Dipp2})_2]OTf$,[4] and $[TlNi(CNAr^{Mes2})_3]OTf$[5] were prepared as previously described. $NaBAr^F_4$ was prepared according to a literature procedure.[6]

Solution ^1H, $^{13}C\{^1H\}$, ^{19}F, and ^{195}Pt NMR spectra were recorded on a Jeol ECA 500, a Varian X-SENS 500, or a Bruker Avance 300 spectrometer. ^1H and $^{13}C\{^1H\}$ chemical shifts are reported in ppm relative to $SiMe_4$ (^1H and ^{13}C δ = 0.0 ppm) with reference to residual solvent resonances of 7.16 ppm (^1H) and 128.06 ppm (^{13}C) for C_6D_6. ^{19}F NMR spectra were referenced externally to neat trifluoroacetic acid, $F_3CC(O)OH$ (δ = −78.5 ppm vs. $CFCl_3$ = 0.0 ppm). ^{195}Pt spectra (direct detection) were referenced externally to 1.2 M $Na_2[PtCl_6]$ in D_2O (δ = 0 ppm). FTIR spectra were recorded on a Thermo-Nicolet iS10 FTIR spectrometer. Samples were prepared as either KBr pellets or as C_6D_6 solutions injected into a ThermoFisher solution cell equipped with KBr windows. For solution FTIR spectra, solvent peaks were digitally subtracted from all spectra by comparison with an authentic spectrum obtained immediately prior to that of the sample. The following abbreviations were used for the intensities and characteristics of important IR absorption bands: vs = very strong, s = strong, m = medium, w = weak, vw = very weak; b = broad, vb = very broad, sh = shoulder. Combustion analyses were performed by Midwest Microlabs of Indianapolis, IN (USA) or Robertson Microlit Laboratories of Madison, NJ (USA).

S1.2. Synthesis of $[TlPt(CNAr^{Dipp2})_2]OTf$ (1). To a solid mixture of $Pt(CNAr^{Dipp2})_2$ (0.100 g, 0.096 mmol) and TlOTf (0.036 g, 0.102 mmol, 1.06 equiv) was added 10 mL Et_2O. This solution was stirred vigorously for 3 h, during which time a yellow precipitate formed. Benzene (1.5 mL) was added to the reaction mixture, which was stirred until no precipitate was visible. Filtration of this solution and storage at –35 ˚C overnight yielded a crop of crystals, which were collected and dried. Yield: 0.088 g, 0.063 mmol, 66%. ^1H NMR (C_6D_6, 499.8 MHz, 20 ˚C): δ = 7.42 (t, 4H, J = 8 Hz, *p*-Dipp), 7.27 (d, 8H, J = 8 Hz, *m*-Dipp), 6.87 (t, 2H, J = 8 Hz, p-Ar^{Dipp2}), 6.80 (d, 4H, J = 8 Hz, m-Ar^{Dipp2}), 2.50 (septet, 8H, J = 7 Hz, $CH(CH_3)_2$), 1.24 (d, 24H, J = 7 Hz, $CH(CH_3)_2$), 1.06 (d, 24H, J = 7 Hz, $CH(CH_3)_2$) ppm. $^{13}C\{^1H\}$ NMR (C_6D_6, 125.7 MHz, 20 ˚C): δ = 159.8 (*C*≡N), 147.1, 139.6, 134.4, 130.4, 129.9, 129.4, 126.9, 123.9, 31.5, 24.4, 24.4 ppm. ^{19}F NMR (C_6D_6, 282.3 MHz, 20 ˚C): δ = –78.2 (s, OTf) ppm. FTIR (C_6D_6, 20 ˚C): υ(C≡N) = 2112 cm^{-1} (vs), also 3063, 2963, 2927, 2868, 1461, 1413, 1385, 1362, 1294, 1232, 1220, 1163, 1056, 1023, 805, 793, 758, 636 cm^{-1}. Anal. calcd for $C_{63}H_{74}N_2F_3O_3SPtTl$: C, 54.21; H, 5.34; N, 2.01. Found: C, 54.30; H, 5.07; N, 2.01.

S1.3. Synthesis of [TlPt(CNAr^{Dipp2})$_2$]BAr$^F_4$ (3). To an Et_2O solution of [TlPt(CNAr^{Dipp2})$_2$]OTf (0.096 g, 0.069 mmol, 5 mL) was added an Et_2O solution of NaBAr$^F_4$ (0.062 g, 0.070 mmol, 1.01 equiv, 3 mL). The solution was allowed to stir for 3 h, during which time NaOTf precipitated from solution. After filtration of this solution through Celite, all volatiles were removed *in vacuo*. Storage of a 2:1 Et_2O/(TMS)$_2$O solution (1.5 mL) at –35 ˚C overnight provided yellow crystals, which were collected and dried *in vacuo*. Yield: 0.100 g, 0.047 mmol, 68%. Crystals grown by this method correspond to **3**(Et_2O). Crystals of solvent-free **3** suitable for X-ray diffraction were grown from a toluene solution stored at –35 ˚C. ^{1}H NMR (C_6D_6, 499.8 MHz, 20 ˚C): δ = 8.39 (br s, 8H, *o*-BAr^F), 7.74 (br s, 4H, *p*-BAr^F), 7.14 (t, 4H, J = 8 Hz, *p*-Dipp), 7.07 (d, 8H, J = 8 Hz, *m*-Dipp), 6.98 (t, 2H, J = 8 Hz, *p-Ar*^{Dipp2}), 6.84 (d, 4H, J = 8 Hz, *m-Ar*^{Dipp2}), 2.42 (septet, 8H, J = 7 Hz, C*H*(CH$_3$)$_2$), 1.09 (d, 24H, J = 7 Hz, CH(C*H*$_3$)$_2$), 1.02 (d, 24H, J = 7 Hz, CH(C*H*$_3$)$_2$) ppm. ^{13}C{^{1}H} NMR (C_6D_6, 125.7 MHz, 20 ˚C): δ = 162.8 (q, $^1J_{C,B}$ = 50 Hz, *ipso*-BAr^F), 156.7 (*C*≡N), 147.6, 139.4, 135.6 (br s, *o*-BAr^F), 134.5, 130.7, 130.5, 130.2, 130.0 (qq, $^2J_{C,F}$ = 32 Hz, $^4J_{C,F}$ = 2 Hz, *m*-BAr^F), 125.8, 125.4 (q, $^1J_{C,F}$ = 272 Hz, -CF$_3$), 123.7, 118.1 (br s, *p*-BAr^F), 31.4, 24.4, 24.0 ppm. ^{19}F NMR (C_6D_6, 282.3 MHz, 20 ˚C): δ = –62.9 (s, C*F*$_3$) ppm. ^{195}Pt NMR (107.5 MHz, C_6D_6, 20 ˚C): –3802 (d, $J_{Pt,Tl}$ = 11.2 kHz) ppm. FTIR (C_6D_6, 20 ˚C): υ(C≡N) = 2121 cm^{-1} (vs), also 3067, 2967, 2929, 2867, 1577, 1460, 1385, 1355, 1277, 1180, 1160, 1127, 1102, 1055, 886, 836, 805, 791, 761, 714, 680, 669 cm^{-1}. Anal. calcd. for $C_{94}H_{86}N_2BF_{24}PtTl$: C, 53.51; H, 4.11; N, 1.33. Found: C, 52.82; H, 4.27; N, 1.26.

S1.4. Synthesis of [TlPd(CNAr^{Dipp2})$_2$]BAr$^F_4$ (4). To an Et_2O solution of [TlPd(CNAr^{Dipp2})$_2$]OTf (0.069 g, 0.053 mmol, 5 mL) was added an Et_2O solution of NaBAr$^F_4$ (0.047 g, 0.053 mmol, 1.00 equiv, 3 mL). This solution was allowed to stir for 24 h, during which time NaOTf precipitated from solution. After filtration of this solution through Celite, all volatiles were removed *in vacuo*. Storage of a 2:1 Et_2O/(TMS)$_2$O solution (1 mL) at –35 ˚C for 3 days yielded orange crystals, which were collected and dried *in vacuo*. Yield: 0.081 g, 0.040 mmol, 58%. Crystals grown by this method correspond to **4**(Et_2O). Crystals of **4**(Tol)$_2$ suitable for X-ray diffraction were grown from a toluene solution stored at –35 ˚C. ^{1}H NMR (C_6D_6, 499.8 MHz, 20 ˚C): δ = 8.43 (br s, 8H, *o*-BAr^F), 7.73 (br s, 4H, *p*-BAr^F), 7.15 (t, 4H, J = 8 Hz, *p*-Dipp), 7.08 (d, 8H, J = 8 Hz, *m*-Dipp), 6.92 (t, 2H, J = 8 Hz, *p-Ar*^{Dipp2}), 6.79 (d, 4H, J = 8 Hz, *m-Ar*^{Dipp2}), 2.43 (septet, 8H, J = 7 Hz, C*H*(CH$_3$)$_2$), 1.11 (d, 24H, J = 7 Hz, CH(C*H*$_3$)$_2$), 1.04 (d, 24H, J = 7 Hz, CH(C*H*$_3$)$_2$) ppm. ^{13}C{^{1}H} NMR (C_6D_6, 125.7 MHz, 20 ˚C): δ = 167.9 (*C*≡N), 162.8 (q, $^1J_{C,B}$ = 50 Hz, *ipso*-BAr^F), 147.5, 139.0, 135.5 (br s, *o*-BAr^F), 134.7, 130.6, 130.3, 130.1, 129.9 (qq, $^2J_{C,F}$ = 32 Hz, $^4J_{C,F}$ = 2 Hz, *m*-BAr^F), 126.5, 125.3 (q, $^1J_{C,F}$ = 272 Hz, -CF$_3$), 123.5, 118.1 (septet, $^3J_{C,F}$ = 4 Hz, *p*-BAr^F), 31.4, 24.5, 23.9 ppm. ^{19}F NMR (C_6D_6, 282.3 MHz, 20 ˚C): δ = –62.8 (s, -CF$_3$) ppm. FTIR (C_6D_6, 20 ˚C): υ(C≡N) = 2116 cm^{-1} (vs), also 3063, 2964, 2927, 2869, 1464, 1353, 1278, 1178, 1163, 1128, 1106, 1097, 1055, 935, 886, 840, 794, 761, 744, 714, 682, 670 cm^{-1}. Anal. calc'd for $C_{94}H_{86}N_2BF_{24}PdTl$: C, 55.86; H, 4.29; N, 1.39. Found: C, 55.84; H, 4.36; N, 1.33.

S1.5. Synthesis of [AgPt(CNAr^{Dipp2})$_2$]OTf (5). To a solid mixture of Pt(CNAr^{Dipp2})$_2$ (0.100 g, 0.096 mmol) and AgOTf (0.026 g, 0.102 mmol, 1.06 equiv) was added 5 mL Et_2O. The solution was stirred vigorously for 2 h in the dark, during which time a yellow precipitate formed. The mother liquor was decanted off from the solid, which was washed once with Et_2O (3 mL) and dried. The mother liquor was dried *in vacuo*, and the resulting residue was taken up in 1.5 mL of 2:1 Et_2O/C_6H_6. Storage of this solution at –35 ºC overnight yielded a crop of crystals, which

were also collected and dried. Yield: 0.105 g, 0.081 mmol, 84%. Crystals grown by this method correspond to **5**(C_6H_6). Crystals of **5**(THF) suitable for X-ray diffraction were grown from a 1:1 THF/$(TMS)_2O$ solution stored at –35 ˚C. ^{1}H NMR (C_6D_6, 499.8 MHz, 20 ˚C): δ = 7.61 (br t, 4H, *J* = 7 Hz, *p*-Dipp), 7.45 (br d, 8H, *J* = 8 Hz, *m*-Dipp), 6.89 (t, 2H, *J* = 7 Hz, *p-Ar*^{Dipp2}), 6.82 (d, 4H, *J* = 8 Hz, *m-Ar*^{Dipp2}), 2.52 (septet, 8H, J = 7 Hz, $CH(CH_3)_2$), 1.23 (d, 24H, *J* = 7 Hz, $CH(CH_3)_2$), 1.10 (d, 24H, *J* = 7 Hz, $CH(CH_3)_2$) ppm. $^{13}C\{^1H\}$ NMR (C_6D_6, 125.7 MHz, 20 ˚C): δ = 162.4 (*C*≡N), 147.3, 139.4, 134.7, 129.9, 129.5, 129.0, 127.6, 123.831.5, 24.5, 24.3 ppm. ^{19}F NMR (C_6D_6, 282.3 MHz, 20 ˚C): δ = –77.7 (s, OTf) ppm. FTIR (C_6D_6, 20 ˚C): υ(C≡N) = 2094 cm^{-1} (vs), also 3091, 3071, 3036, 2963, 2926, 2869, 1577, 1463, 1412, 1385, 1363, 1304, 1250, 1234, 1217, 1163, 1056, 1025, 812, 793, 760, 680, 637 cm^{-1}. Anal. calcd. for $C_{63}H_{74}N_2F_3O_3SAgPt$: C, 58.24; H, 5.74; N, 2.16. Found: C, 58.50; H, 5.85; N, 2.19.

S1.6. Synthesis of [AgPd(CNAr^{Dipp2})$_2$]OTf (6). To a thawing Et_2O solution of Pt(CNAr^{Dipp2})$_2$ (0.100 g, 0.105 mmol, 4 mL) was added an equally cold 1:1 Et_2O/THF solution of AgOTf (0.028 g, 0.109 mmol, 1.04 equiv, 2 mL). The solution was stirred vigorously while keeping the temperature near the freezing point of the solvent, during which time a pale yellow precipitate formed. After 30 min, this precipitate was isolated via filtration over a fritted funnel and dried, affording [AgPd(CNAr^{Dipp2})$_2$]OTf as an analytically pure pale yellow powder. Yield: 0.044 g, 0.036 mmol, 35%. Crystals of **6**(THF) were grown from a 1:1 THF/$(TMS)_2O$ solution stored at –35 ˚C overnight. Crystals of **6**(C_7H_8) were grown from a toluene solution layered with *n*-hexane and stored at –35 ˚C. ^{1}H NMR (C_6D_6, 499.8 MHz, 20 ˚C): δ = 7.53 (br t, 4H, *J* = 7 Hz, *p*-Dipp), 7.41 (br d, 8H, *J* = 8 Hz, *m*-Dipp), 6.89 (t, 2H, *J* = 7 Hz, *p-Ar*^{Dipp2}), 6.84 (d, 4H, *J* = 8 Hz, *m-Ar*^{Dipp2}), 2.55 (septet, 8H, J = 7 Hz, $CH(CH_3)_2$), 1.27 (d, 24H, *J* = 7 Hz, $CH(CH_3)_2$), 1.11 (d, 24H, *J* = 7 Hz, $CH(CH_3)_2$) ppm. $^{13}C\{^1H\}$ NMR (C_6D_6, 125.7 MHz, 20 ˚C): δ = 158.9 (*C*≡N), 147.3, 139.3, 135.1, 130.0, 129.3, 128.4, 123.8, 31.5, 24.6, 24.3 ppm. *Note: One C_{aryl} resonance is believed to be overlapped by the resonances of the C_6D_6 solvent.* ^{19}F NMR (C_6D_6, 282.3 MHz, 20 ˚C): δ = –77.6 (s, OTf) ppm. FTIR (C_6D_6, 20 ˚C): υ(C≡N) = 2082 cm^{-1} (vs), also 3063, 3025, 2963, 2926, 2868, 2850, 1595, 1578, 1460, 1413, 1384, 1363, 1301, 1235, 1218, 1205, 1162, 1035, 1026, 792, 759, 731, 696, 636 cm^{-1}. Anal. calcd. for $C_{63}H_{74}N_2F_3O_3SAgPd$: C, 62.50; H, 6.16; N, 2.31. Found: C, 61.92; H, 6.05; N, 2.27.

S1.7. Synthesis of {[Ag$_2$Pt(CNAr^{Dipp2})$_2$(η^1-C$_6$H$_6$)]$_2$(μ-OTf)$_2$}(OTf)$_2$ (7). To a THF solution of [AgPt(CNAr^{Dipp2})$_2$]OTf (0.081 g, 0.062 mmol, 2 mL) was added a THF solution of AgOTf (0.024 mmol, 0.094 mmol, 1.50 equiv, 2 mL). This solution was stirred in the dark for 30 min, whereupon all volatiles were removed in vacuo. Addition of 3 mL C_6H_6 to this residue resulted in precipitation of a yellow solid after a few minutes of stirring. This precipitate was isolated via decantation, washed with additional C_6H_6 (3 mL), and dried *in vacuo*. Yield: 0.060 g, 0.019 mmol, 62%. Crystals suitable for X-ray diffraction were grown from a dilute 20:1 C_6H_6/THF solution at room temperature. As **7** is only soluble in coordinating solvents, which outcompete Pt for ligation of Ag, NMR data were not obtained. FTIR (KBr pellet, 20 ˚C): υ(C≡N) = 2132 cm^{-1} (vs) and 2169 (m sh), also 3089, 3065, 3034, 2962, 2928, 2870, 1617, 1593, 1576, 1466, 1412, 1385, 1364, 1289, 1276, 1262, 1240, 1220, 1170, 1152, 1056, 1043, 1029, 1020, 832, 825, 807, 796, 772, 762, 714, 688, 636, 625 cm^{-1}. Anal. calcd. for $C_{128}H_{148}N_4F_{12}O_{12}S_4Pt_2Ag_4$: C, 49.39; H, 4.79; N, 1.80. Found: 47.67; H, 5.06; N, 1.40. Repeated attempts to obtain a more satisfactory combustion analysis were unsuccessful, a result that we attribute to the light sensitivity exhibited by complex **7**.

S1.8. X-ray Absorption Near-Edge Spectroscopy (XANES). *X-ray Absorption Near-Edge Spectroscopy (XANES)*. X-ray absorption measurements were conducted at the Stanford Synchrotron Radiation Lightsource (SSRL) at beamlines 7-3 and 9-3. At both beam lines, a Si(220) double-crystal monochromator was used energy for energy selection, in combination with a harmonic rejection mirror. Incident and transmitted X-ray intensities were monitored using Ar- (at the Pd K-edge) or N_2- (at the Ni K-edge) filled ionization chambers. X-ray absorption was measured in transmittance mode. During data collection, samples were maintained at a temperature of approximately 10 K using an Oxford Instruments liquid helium flow cryostat. Internal energy calibrations were performed by simultaneous measurement of Pd and Ni reference foils placed between the second and third ionization chamber, with the inflection point assigned at 24350 eV and 8333.0 eV for the first inflection point of the Pd and Ni foils, respectively. Data represent 2–5 scan averages and were processed by fitting a second-order polynomial to the pre-edge region and subtracting this background from the entire spectrum. A spline was used to model the smooth background above the edge. The data were normalized by subtracting the spline and normalizing the postedge to 1.0.

S2. CSD Search Results

S2.1. General. The Cambridge Structural Database[7–9] was queried using the CSD Conquest software. The desired metrical parameters were plotted using the CSD Legacy Vista program. All results that were returned for a given set of search constraints are displayed in the histograms below (Figures S2.1-4), as are the respective mean and standard deviation values.

S2.2. CSD search criteria and results for structurally characterized compounds containing Pt-O_{Ether} bonds. The search was constrained to compounds containing single bonds between Pt and O, with the oxygen atom additionally containing single bonds to exactly two carbon atoms and no additional atoms. A total of 33 data points were obtained and are plotted in the histogram below.

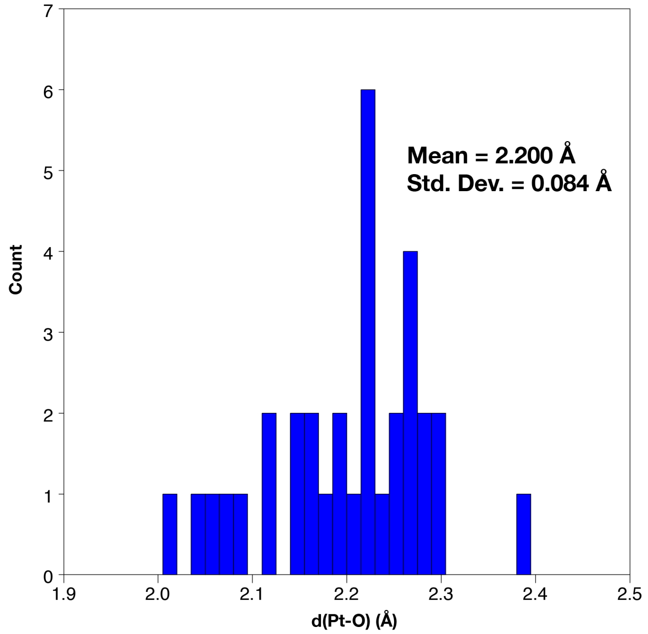

Figure S2.1. CSD search results for complexes containing Pt-O_{Ether} bonds.

S2.3. CSD search criteria and results for structurally characterized compounds containing Pd-O_{Ether} bonds. The search was constrained to compounds containing single bonds between Pd and O, with the oxygen atom additionally containing single bonds to exactly two carbon atoms and no additional atoms. A total of 135 data points were obtained and are plotted in the histogram below.

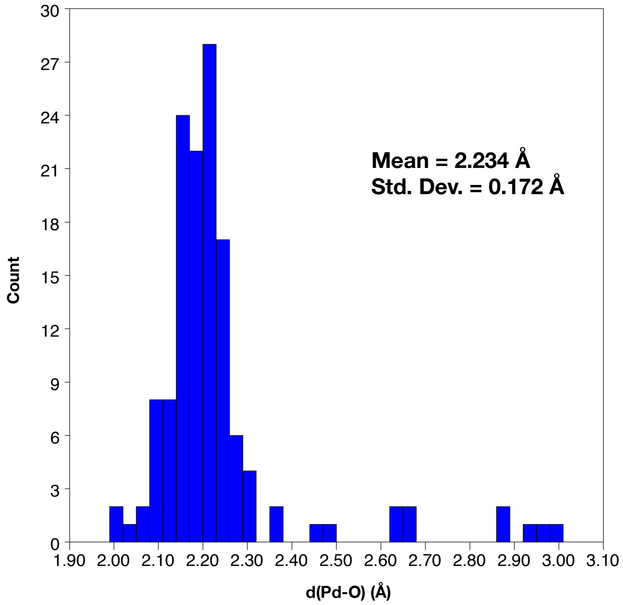

Figure S2.2. CSD search results for complexes containing Pd-O_{Ether} bonds.

S2.4. CSD search criteria and results for structurally characterized compounds containing Pt-Ag bonds. The search was constrained to compounds that were defined as containing a bonding interaction between Pt and Ag. A total of 306 data points were obtained and are plotted in the histogram below.

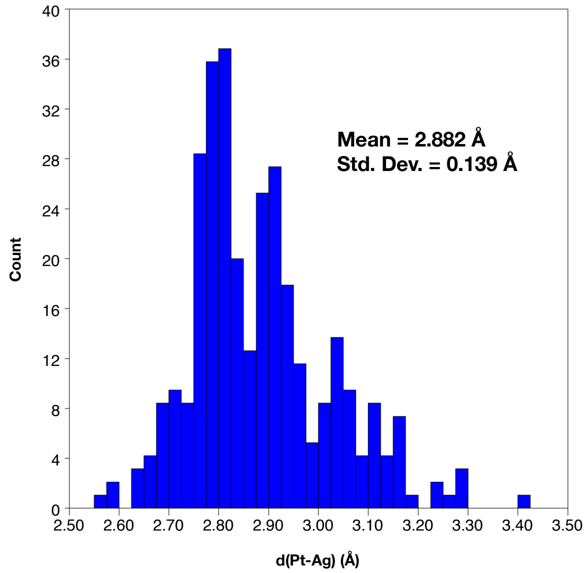

Figure S2.3. CSD search results for complexes containing Pt-Ag bonding interactions.

S2.4. CSD search criteria and results for structurally characterized compounds containing Pd-Ag bonds. The search was constrained to compounds that were defined as containing a bonding interaction between Pd and Ag. A total of 46 data points were obtained and are plotted in the histogram below.

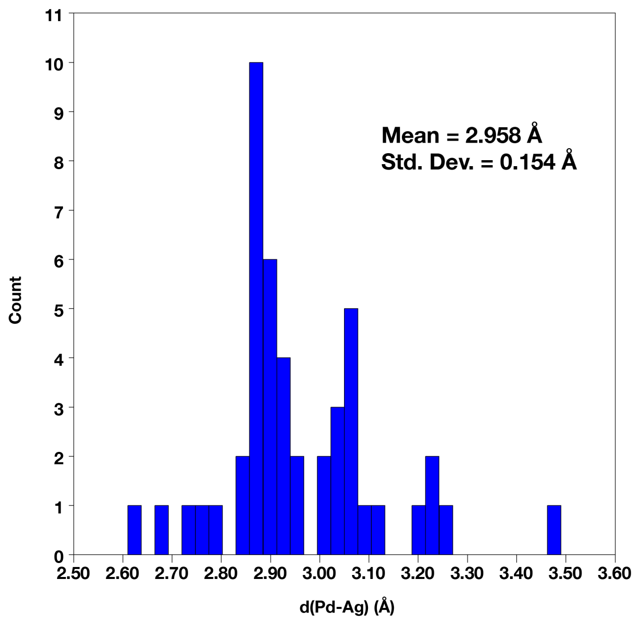

Figure S2.4. CSD search results for complexes containing Pd-Ag bonding interactions.

S3. Crystallographic Structure Determinations

S3.1. General. Single crystal X-ray structure determinations were carried out at low temperature on Bruker Kappa diffractometers equipped with a Mo radiation source and a Bruker APEX or APEX-II detector. All structures were solved via direct methods with SHELXS[10] and refined by full-matrix least-squares procedures using SHELXL[10] within the Olex2 small-molecule solution, refinement and analysis software.[11] Crystallographic data collection and refinement information are listed in Table S3.1.

S3.2. Information on Crystallographic Disorder. The following molecules contained positionally disordered components that were modeled and refined anisotropically. They are listed along with their respective disordered components:

$[(Et_2O)TlPt(CNAr^{Dipp2})_2]BAr^F_4$ (**3**(Et_2O)): Two-site positional disorder of one Tl atom and its coordinated diethyl ether; two-site positional disorder of the other Tl-bound diethyl ether; two-site disorder of one Dipp ring; two-site disorder of several isopropyl groups; rotational disorder of several –CF_3 groups.

$[TlPt(CNAr^{Dipp2})_2]BAr^F_4$ (**3**): Two-site disorder of one isopropyl group; rotational disorder of several –CF_3 groups.

$[(Et_2O)TlPd(CNAr^{Dipp2})_2]BAr^F_4$ (**4**(Et_2O)): Two-site positional disorder of one Tl atom and its coordinated diethyl ether; rotational disorder of several –CF_3 groups.

$[(\eta^6\text{-}C_7H_8)_2TlPd(CNAr^{Dipp2})_2]BAr^F_4$ (**4**($Tol)_2$): Two-site disorder of one Tl-bound toluene; rotational disorder of several –CF_3 groups.

$[AgPt(CNAr^{Dipp2})_2(\eta^1\text{-}C_6H_6)]OTf$ (**5**(C_6H_6)): Two-site disorder of triflate anion; two-site disorder of one isopropyl group.

$[AgPt(CNAr^{Dipp2})_2(THF)]OTf$ (**5**(THF)): Two-site disorder of the Pt-bound molecule of THF.

$\{[Ag_2Pt(CNAr^{Dipp2})_2(\eta^1\text{-}C_6H_6)]_2(\mu\text{-}OTf)_2\}(OTf)_2$ (**7**): Two-site disorder of one bridging triflate anion; two-site disorder of one co-crystallized molecule of benzene.

In addition, the following molecules contained co-crystallized solvent molecules that were severely disordered and could not be properly modeled. The Platon routine SQUEEZE[12] was used to account for these disordered molecules as a diffuse contribution to the overall scattering pattern without specific atom positions:

$[TlPt(CNAr^{Dipp2})_2]OTf$ (**1**): Two molecules of diethyl ether per unit cell.

$[AgPt(CNAr^{Dipp2})_2(THF)]OTf$ (**5**(THF)): Twelve molecules of THF per unit cell.

$[AgPd(CNAr^{Dipp2})_2(THF)]OTf$ (**6**(THF)): Twenty-six molecules of THF per unit cell.

S3.3. CCDC Deposition. All crystal structures reported herein have been deposited with the Cambridge Crystallographic Data Center (CCDC). They have been assigned the following CCDC deposition numbers:

1414413 (Compound **4**(Et_2O))
1414414 (Compound **4**($(Tol)_2$))
1414415 (Compound **7**)
1414416 (Compound **5**(C_6H_6)
1414417 (Compound **1**)
1414418 (Compound **6**(THF))
1414419 (Compound **5**(THF))
1414420 (Compound **6**(C_7H_8))
1414421 (Compound **3**(Et_2O))
1414422 (Compound **3**)

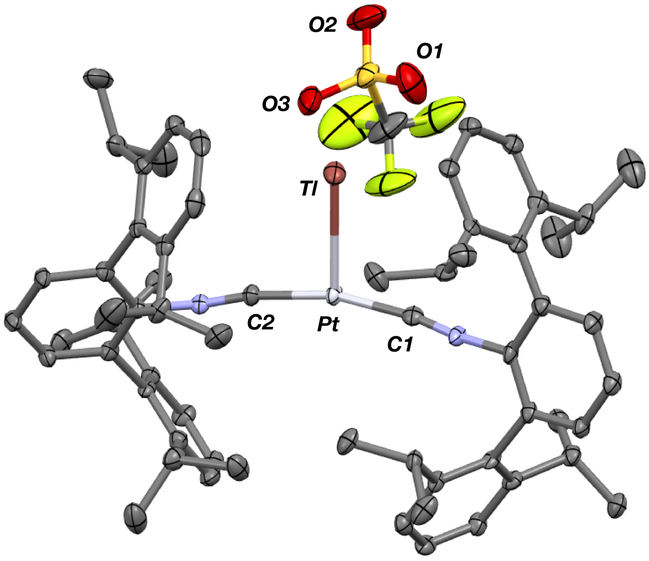

Figure S3.1. Molecular structure of [TlPt(CNAr^{Dipp2})$_2$]OTf (**1**). Selected bond distances (Å) and angles (º): Pt-Tl = 2.8617(3); Tl-O3 = 2.799(5); Pt-C1 = 1.919(5); Pt-C2 = 1.916(5); C1-Pt-Tl = 95.9(1); C2-Pt-Tl = 95.9(1); C1-Pt-C2 = 165.3(2).

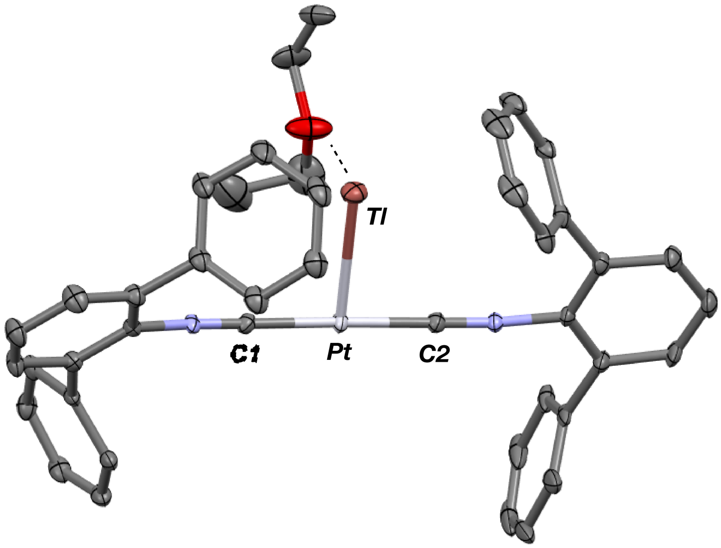

Figure S3.2. Molecular structure of [(Et$_2$O)TlPt(CNAr^{Dipp2})$_2$]BAr$^F{}_4$ (**3**(Et$_2$O)). Isopropyl groups and BAr$^F{}_4$ counteranion have been omitted for clarity. Only one of two crystallographically independent molecules is shown, with metrical parameters reported as averages between these molecules. Selected bond distances (Å) and angles (º): Pt-Tl = 2.7710(4); Pt-C1 = 1.932(4); Pt-C2 = 1.914(4); Tl-O = 2.760(3); C1-Pt-Tl = 94.5(1); C2-Pt-Tl = 86.2(1); C1-Pt-C2 = 173.4(1).

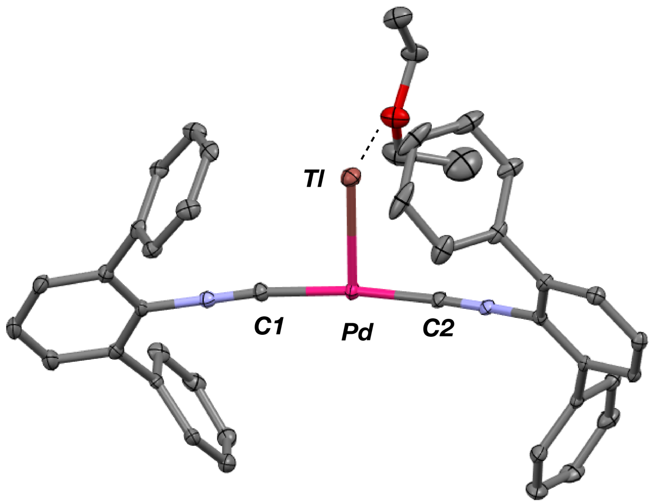

Figure S3.3. Molecular structure of [(Et$_2$O)TlPd(CNAr^{Dipp2})$_2$]BAr$^F_4$ (**4**(Et$_2$O)). Isopropyl groups and BAr$^F_4$ counteranion have been omitted for clarity. Only one of two crystallographically independent molecules is shown, with metrical parameters reported as averages between these molecules. Selected bond distances (Å) and angles (º): Pd-Tl = 2.7481(5); Pd-C1 = 1.964(4); Pd-C2 = 1.953(5); Tl-O = 2.729(3); C1-Pd-Tl = 93.8(2); C2-Pd-Tl = 85.2(1); C1-Pd-C2 = 171.2(2).

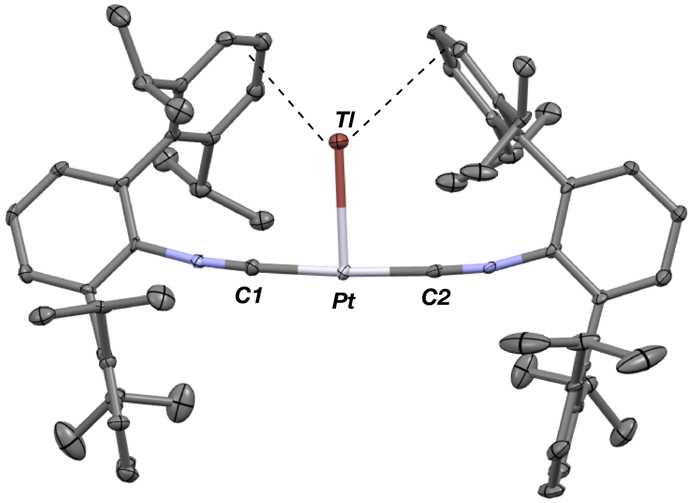

Figure S3.4. Molecular structure of [TlPt(CNAr^{Dipp2})$_2$]BAr$^F_4$ (**3**). BAr$^F_4$ counterion has been omitted for clarity. Only one of two crystallographically independent molecules is shown, with metrical parameters reported as averages between these molecules. Selected bond distances (Å) and angles (º): Pt-Tl = 2.7659(5); Pt-C1 = 1.933(8); Pt-C2 = 1.934(8); C1-Pt-Tl = 93.2(2); C2-Pt-Tl = 86.6(2); C1-Pt-C2 = 173.8(3).

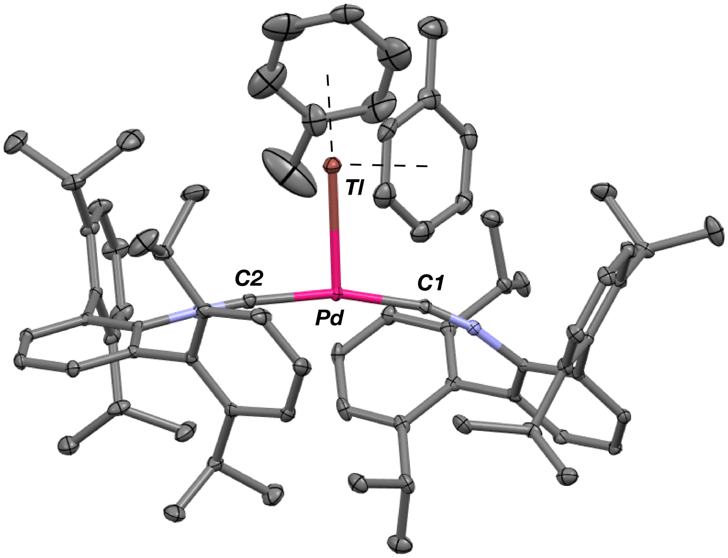

Figure S3.5. Molecular structure of $[(\eta^6\text{-}C_7H_8)_2TlPd(CNAr^{Dipp2})_2]BAr^F{}_4$ (**4**(Tol)$_2$). $BAr^F{}_4$ counterion has been omitted for clarity. Selected bond distances (Å) and angles (º): Pd-Tl = 2.7770(2); Pd-C1 = 1.960(3); Pd-C2 = 1.969(3); C1-Pd-Tl = 88.34(7); C2-Pd-Tl = 104.32(7); C1-Pd-C2 = 166.5(1).

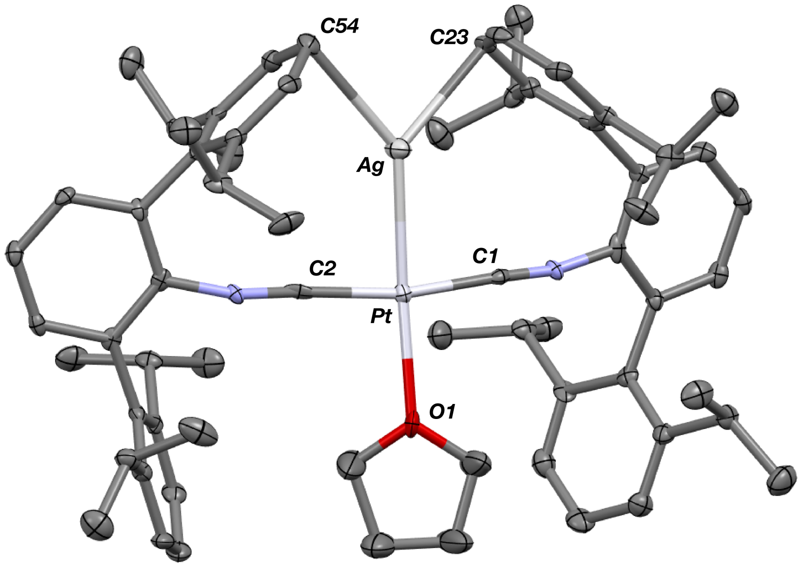

Figure S3.6. Molecular structure of $[AgPt(CNAr^{Dipp2})_2(THF)]OTf$ (**5**(THF)). Triflate counteranion has been omitted for clarity. Selected bond distances (Å) and angles (º): Pt-Ag = 2.6299(6); Pt-C1 = 1.932(8); Pt-C2 = 1.920(9); Pt-O1 = 2.366(5); Ag-C23 = 2.661(8); Ag-C54 = 2.660(8); C1-Pt-Ag = 83.9(2); C2-Pt-Ag = 86.4(2); O1-Pt-Ag = 173.0(1).

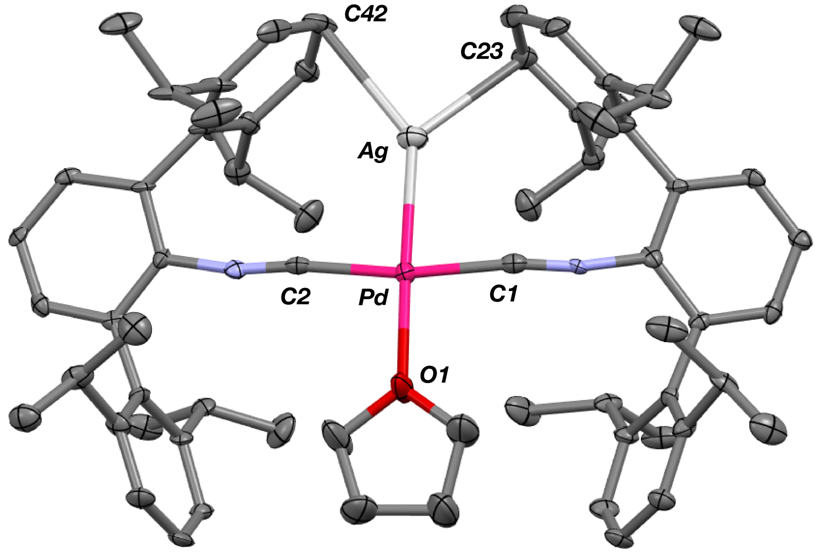

Figure S3.7. Molecular structure of [AgPd(CNAr^{Dipp2})$_2$(THF)]OTf (**6**(THF)). Triflate counteranion has been omitted for clarity. Selected bond distances (Å) and angles (º): Pd-Ag = 2.6303(9); Pd-C1 = 1.94(1); Pd-C2 = 1.941(9); Pd-O1 = 2.326(7); Ag-C23 = 2.705(9); Ag-C42 = 2.724(9); C1-Pd-Ag = 84.7(2); C2-Pd-Ag = 80.9(2); O1-Pd-Ag = 175.4(2).

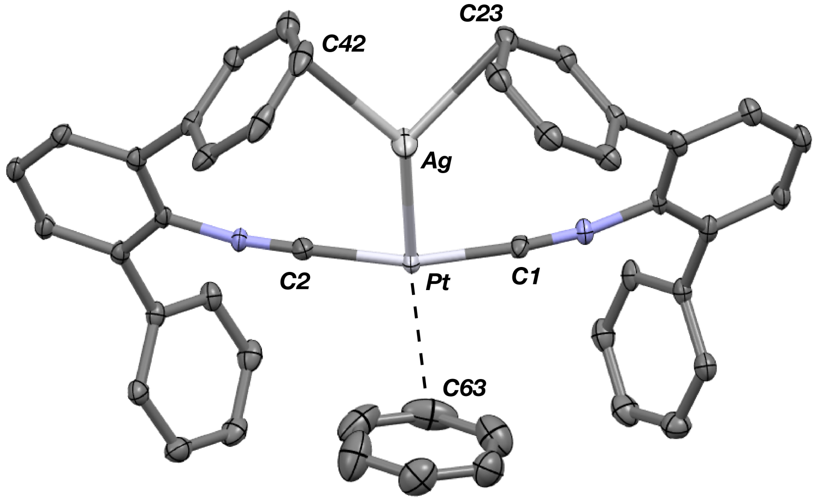

Figure S3.8. Molecular structure of [AgPt(CNAr^{Dipp2})$_2$(η^1-C_6H_6)]OTf (**5**(C_6H_6)). Isopropyl groups and triflate counteranion have been omitted for clarity. Selected bond distances (Å) and angles (º): Pt-Ag = 2.6463(5); Pt-C1 = 1.923(5); Pt-C2 = 1.909(4); Pt-C63 = 2.885(7); Ag-C23 = 2.587(5); Ag-C42 = 2.633(5); C1-Pt-Ag = 86.7(2); C2-Pt-Ag = 84.9(1).

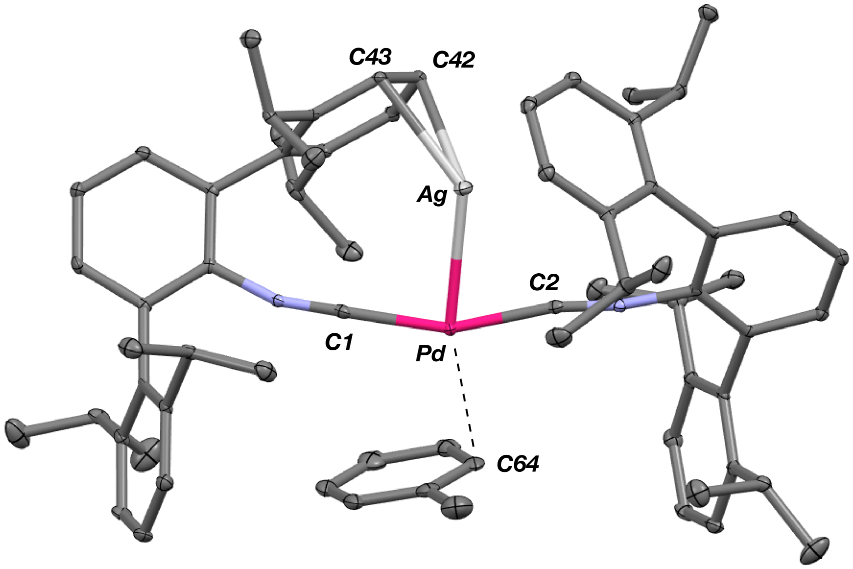

Figure S3.9. Molecular structure of [AgPd(CNAr^{Dipp2})$_2$(η^1-C_7H_8)]OTf (**6**(C_7H_8)). Triflate counteranion has been omitted for clarity. Selected bond distances (Å) and angles (º): Pd-Ag = 2.6112(4); Pd-C1 = 1.962(3); Pd-C2 = 1.939(3); Pd-C64 = 2.496(3); Ag-C42 = 2.632(2); Ag-C43 = 2.506(2); C1-Pd-Ag = 86.88(7); C2-Pd-Ag = 72.49(7).

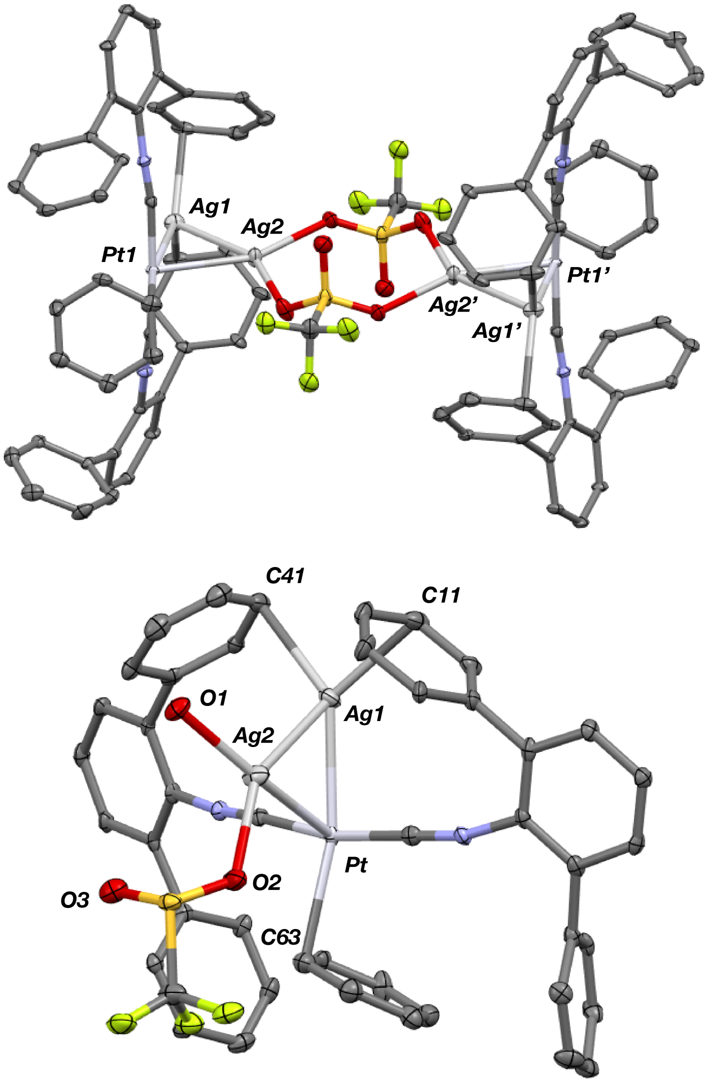

Figure S3.10. Top: Molecular structure $\{[Ag_2Pt(CNAr^{Dipp2})_2(\eta^1\text{-}C_6H_6)]_2(\mu\text{-}OTf)_2\}(OTf)_2$ (**7**), with isopropyl groups and two non-coordinating triflate counteranions omitted for clarity. Bottom: Zoom-in on one half of the dimeric structure (other half of dimer is related by a crystallographic inversion center), clearly showing the Pt-η^1-C_6H_6 interaction. Selected bond distances (Å) and angles (º): Pt-Ag1 = 2.6799(7); Pt-Ag2 = 2.6888(7); Ag1-Ag2 = 2.7684(8); Pt-C63 = 2.529(7); Ag1-C11 = 2.490(7); Ag1-C41 = 2.608(7); Pt-Ag1-Ag2 = 59.12(2); Ag1-Ag2-Pt = 58.80(2); Ag2-Pt-Ag1 = 62.08(2).

Table S3.1. Crystallographic data collection and refinement information.

Name	[TlPt(CNAr^{Dipp2})$_2$]OTf • 2 Et$_2$O (**1**•2(Et$_2$O))	[(Et$_2$O)TlPt(CNAr^{Dipp2})$_2$]BAr$^F_4$ (**3**(Et$_2$O))	[(Et$_2$O)TlPd(CNAr^{Dipp2})$_2$] BAr$^F_4$ (**4**(Et$_2$O))
Formula	$C_{71}H_{94}N_2F_3SO_5PtTl$	$C_{98}H_{96}N_2BOF_{24}PtTl$	$C_{98}H_{96}N_2BOF_{24}PdTl$
Crystal System	Triclinic	Triclinic	Triclinic
Space Group	*P*-1	*P*-1	*P*-1
a, Å	13.9500(8)	12.732(1)	12.7422(9)
b, Å	16.277(1)	26.642(3)	26.679(2)
c, Å	17.621(1)	28.428(3)	28.503(2)
α, deg	68.887(3)	99.200(4)	99.098(1)
β, deg	70.018(3)	90.224(4)	90.144(1)
γ, deg	71.465(3)	90.514(4)	90.509(1)
V, Å^3	3379.6(4)	9519(2)	9567(1)
Z	2	4	4
Radiation (λ, Å)	Mo-Kα, 0.71073	Mo-Kα, 0.71073	Mo-Kα, 0.71073
ρ (calcd.), g/cm^3	1.517	1.524	1.455
μ (Mo Kα), mm^{-1}	4.537	3.253	1.963
Temp, K	100	100	100
θ max, deg	25.408	25.474	24.713
data/parameters	12410/683	35166/2838	32575/2560
R_1	0.0344	0.0305	0.0454
wR_2	0.0737	0.0671	0.0803
GOF	1.057	1.010	1.029

Table S3.1. Con't.

	[TlPt(CNAr^{Dipp2})$_2$]BAr$^F_4$ (**3**)	[(η^6-C$_7$H$_8$)$_2$ TlPd(CNAr^{Dipp2})$_2$]BAr$^F_4$ (**4**(Tol)$_2$)	[AgPt(CNAr^{Dipp2})$_2$(THF)]OTf • 3 THF (**5**(THF) • 3 THF)
Formula	$C_{94}H_{86}N_2BF_{24}PtTl$	$C_{108}H_{102}N_2BF_{24}PdTl$	$C_{79}H_{106}N_2F_3O_7SPtAg$
Crystal System	Triclinic	Triclinic	Orthorhombic
Space Group	*P*-1	*P*-1	$P2_12_12_1$
a, Å	14.6265(7)	12.5519(5)	15.648(2)
b, Å	20.654(1)	20.2591(8)	21.250(2)

c, Å	31.199(2)	21.6470(8)	24.077(2)
α, deg	76.597(2)	105.597(1)	90
β, deg	79.378(3)	102.325(1)	90
γ, deg	89.566(3)	97.308(1)	90
V, $Å^3$	9005.2(8)	5079.1(3)	8006(1)
Z	4	2	4
Radiation (λ, Å)	Mo-Kα, 0.71073	Mo-Kα, 0.71073	Mo-Kα, 0.71073
ρ (calcd.), g/cm^3	1.556	1.442	1.312
μ (Mo Kα), mm^{-1}	3.434	1.853	2.071
Temp, K	100	100	100
θ max, deg	25.408	25.549	25.404
data/parameters	32879/2461	18800/1339	13450/741
R_1	0.0601	0.0294	0.0359
wR_2	0.1030	0.0653	0.0851
GOF	1.013	1.029	1.033

Table S3.1. Con't.

Name	$[AgPd(CNAr^{Dipp2})_2(THF)]OTf$ • 6.5 THF (**5**(THF) • 6.5 THF)	$[AgPt(CNAr^{Dipp2})_2(\eta^1\text{-}C_6H_6)]OTf$ •3 C_6H_6 (**5**(C_6H_6) • 3(C_6H_6))	$[AgPd(CNAr^{Dipp2})_2(\eta^1\text{-}C_7H_8)]OTf$ • C_7H_8 (**6**(C_7H_8) • C_7H_8)
Formula	$C_{93}H_{134}N_2O_{10.5}F_3SPdAg$	$C_{81}H_{92}N_2O_3F_3SPtAg$	$C_{84}H_{98}N_2O_3F_3SPdAg$
Crystal System	Orthorhombic	Monoclinic	Triclinic
Space Group	$P2_12_12_1$	$P2_1/n$	*P*-1
a, Å	15.594(2)	18.606(1)	14.529(2)
b, Å	21.315(2)	19.772(1)	15.663(2)
c, Å	24.141(3)	20.951(1)	17.673(2)
α, deg	90	90	96.662(4)
β, deg	90	106.515(2)	109.412(4)
γ, deg	90	90	94.019(4)
V, $Å^3$	8024(1)	7389.4(8)	3742.0(8)
Z	4	4	2
Radiation (λ, Å)	Mo-Kα, 0.71073	Mo-Kα, 0.71073	Mo-Kα, 0.71073

ρ (calcd.), g/cm^3	1.450	1.378	1.320
μ (Mo Kα), mm^{-1}	0.565	2.240	0.586
Temp, K	100	150	100
θ max, deg	25.436	25.448	25.422
data/parameters	14709/728	13613/891	13526/884
R_1	0.0618	0.0419	0.0350
wR_2	0.1691	0.0885	0.0696
GOF	1.069	1.078	1.028

Table S3.1. Con't.

Name	$\{[Ag_2Pt(CNAr^{Dipp2})_2(\eta^1\text{-}C_6H_6)]_2(\mu\text{-}OTf)_2\}(OTf)_2$ (**7**)
Formula	$C_{152}H_{172}N_4O_{12}F_{12}S_4Pt_2Ag_4$
Crystal System	Monoclinic
Space Group	$P2_1/n$
a, Å	11.780(1)
b, Å	29.620(2)
c, Å	20.936(2)
α, deg	90
β, deg	90.390(4)
γ, deg	90
V, $Å^3$	7305(1)
Z	2
Radiation (λ, Å)	Mo-Kα, 0.71073
ρ (calcd.), g/cm^3	1.557
μ (Mo Kα), mm^{-1}	2.566
Temp, K	100
θ max, deg	25.460
data/parameters	13466/953
R_1	0.0503
wR_2	0.0810
GOF	1.003

S4. References

1. W. L. F. Armarego and C. L. L. Chai, *Purification of Laboratory Chemicals,* 5th Ed.; Elsevier, 2003.
2. A. B. Pangborn, M. A. Giardello, R. H. Grubbs, R. K. Rosen, and F. J. Timmers, *Organometallics,* 1996, **15**, 1518–1520.
3. D. L. Reger, T. D. Wright, C. A. Little, J. J. S. Lambda, and M. D. Smith, *Inorg. Chem.,* 2001, **40**, 3810–3814.
4. B. R. Barnett, C. E. Moore, A. L. Rheingold, and J. S. Figueroa, *J. Am. Chem. Soc.,* 2014, **136**, 10262–10265.
5. L. A. Labios, M. D. Millard, A. L. Rheingold, and J. S. Figueroa, *J. Am. Chem. Soc.,* 2009, **131**, 11318–11319.
6. B. J. Fox, M. D. Millard, A. G. DiPasquale, A. L. Rheingold, and J. S. Figueroa, *Angew. Chem., Int. Ed.,* 2009, **48**, 3473–3477.
7. Cambridge Structural Database (CSD), version 5.36 (May 2015 update).
8. F. H. Allen, *Acta Crystallogr. B.,* 2002, **58**, 380.
9. F. H. Allen and W. D. S. Motherwell, *Acta Crystallogr. B.,* 2002, **58**, 407.
10. G. M. Sheldrick, *Acta Crystallogr. A.,* 2008, **64**, 112.
11. O. V. Dolomanov, L. J. Bourhis, R. J. Gildea, J. A. K. Howard, and H. Puschmann, *J. Appl. Cryst.* 2009, **42**, 339.
12. P. van der Sluis and A. L. Spek, *Acta Crystallogr.*, 1990, **A46**, 194.
